# Tree diversity and soil fertility interactions drive carbon storage in degraded cocoa landscapes of Côte d’Ivoire

**DOI:** 10.3389/fpls.2025.1731574

**Published:** 2025-12-16

**Authors:** Alain R. Atangana, Guillaume Kouassi Koffi, Kouassi Bruno Kpangui, Ebagnerin Jerôme Tondoh, Valentin L. F. Wolf, Christophe Kouamé, Damase Khasa

**Affiliations:** 1 CIFOR-ICRAF Cote d’Ivoire Country Programme, Abidjan, Côte d’Ivoire; 2 Unité de Formation et de Recherche (UFR) Environnement, Université Jean Lorougnon Guédé, Daloa, Côte d’Ivoire; 3 Université Nangui Abrogoua, Unité de Formation et de Recherche (UFR) des Sciences de la Nature, Abidjan, Côte d’Ivoire; 4 Centre d’étude de la forêt, Institut de biologie intégrative et des systèmes, Université Laval, Quebec City, QC, Canada

**Keywords:** aboveground carbon, cocoa agroforestry, biodiversity–ecosystem function, climate gradients, land-use systems, soil fertility, tropical landscape restoration

## Abstract

**Introduction:**

Understanding the ecological functioning of degraded cocoa landscapes is critical for restoring productivity and ecosystem services in West Africa. This study investigated how climate, geography, and land-use systems interact to shape tree diversity, soil fertility, and aboveground carbon (AGC) stocks across six cocoa-producing sites in Côte d’Ivoire.

**Methods:**

Field inventories and soil sampling to 50 cm depth were conducted across gradients of climate (1,150–1,650 mm rainfall) and land-use systems (fallow, cocoa agroforestry, and monocropping). Mixed effects ANOVA, regression, and redundancy analyses were applied to test three hypotheses linking environmental gradients, tree diversity, and soil–carbon relationships.

**Results:**

Land-use system had the strongest effect on tree diversity (P< 0.0001), with fallows supporting significantly higher richness (7.2 ± 0.51) and Shannon diversity (1.7 ± 0.05) than agroforestry or monocrop systems. Climate and site nested within climate also significantly influenced species abundance and evenness. Soil fertility indicators were primarily controlled by climate and geographic site, with significant effects on pH, exchangeable bases, and structural stability. Interactions between climate and land-use significantly affected soil pH, organic carbon, calcium, and cation exchange capacity. Stepwise regression identified aluminum, potassium, boron, and magnesium as key soil predictors of AGC (R² = 0.14; P< 0.0001), while species richness was the only diversity metric significantly associated with AGC (R² = 0.12; P< 0.0001).

**Discussion:**

These results highlight the dominant role of species richness over evenness and abundance in sustaining soil fertility and carbon storage, emphasizing biodiversity conservation as a cornerstone of cocoa landscape restoration.

## 1 Introduction

Tropical deforestation continues to shape global ecosystems, driving persistent biodiversity losses, declines in soil fertility, and large transfers of carbon from terrestrial pools to the atmosphere (Giam et al., 2017; Faria et al., 2023; Qu et al., 2024). In West Africa, particularly in Côte d’Ivoire, the conversion of moist tropical forests to perennial commodity landscapes—most notably cocoa (*Theobroma cacao*) monocultures—has been a principal driver of these changes (IFFN, 2021). Forest cover in Côte d’Ivoire declined from 16 million hectares in 1960 to 2.97 million hectares by 2020 (IFFN, 2021). Large-scale deforestation in cocoa-producing zones has contributed to reductions in soil organic matter and nutrient stocks, underlying long-term productivity declines in cocoa systems (Schneider et al., 2017; Bouadou Félix et al., 2024). These dynamics create a feedback loop in which declining crop yields encourage further forest clearing, undermining livelihoods and ecosystem services at the landscape scale (Qu et al., 2024; Giam, 2017).

Research indicates that converting forests to tree-crop plantations commonly reduces soil organic carbon (SOC) and total nitrogen in surface soils and alters nutrient cycles unfavorably for sustained crop production unless management compensates for these losses (van Noordwijk et al., 1997; van Straaten et al., 2015; Widyati et al., 2022; Maitra et al., 2024; Qu et al., 2024). In lowland tropical systems, initial SOC losses can be substantial, impacting soil structure, water retention, and nutrient availability (Bouwman, 1989; Veldkamp, 1994; van Noordwijk et al., 1997; Guillaume et al., 2015). These patterns highlight the need to consider soil depth, time since conversion, and management history when evaluating soil consequences of forest-to-cocoa transitions (van Straaten et al., 2015; Qu et al., 2024).

Agroforestry, defined here as the deliberate integration of trees with crops or animals, has been promoted to restore biodiversity and soil functions while maintaining or enhancing farmer incomes (Montagnini, 2020; Castle et al., 2021; Fahad et al., 2022; Leakey and Harding, 2025). Traditional cocoa agroforestry systems retain varying proportions of remnant and planted shade trees, contributing litter and root inputs, modifying microclimate and hydrology, and providing additional products (timber, fruit, fodder) that diversify farmer income and reduce pressure on remnant forests (Akinyele and Donald-Amaeshi, 2021; Lin, 2007; de Carvalho et al., 2021). Meta-analyses and field studies indicate that agroforestry can increase SOC and other soil health indicators relative to monocultures, although effects vary by system type, tree species composition, stand age, and climate (Sanou et al., 2025; Kouadio et al., 2025; Anthelme et al., 2024).

The mechanisms by which trees influence soil fertility and carbon are trait-dependent. Species differ in litter quality, root architecture, mycorrhizal associations, nutrient resorption, and decomposition rates. These traits influence nutrient release timing and stoichiometry, soil organic matter formation and stabilization, and root-derived carbon distribution. Consequently, species identity and functional composition often exert stronger effects on SOC and nutrient pools than species richness per se (Chen et al., 2023; Haghverdi and Kooch, 2019; de la Fuente Cantó et al., 2020).

Empirical studies show mixed results regarding tree diversity effects on soil fertility and carbon. Multispecies plantings can increase aboveground carbon relative to monocultures, particularly over decades (Chen et al., 2023; Schnabel et al., 2025). Soil responses, however, are variable: some studies report increased SOC and nutrient accrual with diversity, while others find no consistent effect or declines in specific elements under certain species mixes (Guo et al., 2019; Haghverdi and Kooch, 2019; Losada et al., 2023; Wartenberg et al., 2017). Recent studies on Oxisols in Central Africa (Cameroon) confirm that integrating cocoa with forest and fruit species increases microbial diversity and abundance (e.g., Actinomycetes, Azotobacter). Ntsoli et al. (2025) show that this enhanced microbial activity is a driver for improved fertility and the maintenance of SOC, ultimately resulting in higher yields. Koné and Yao (2021), working in central west Côte d’Ivoire, demonstrated that mixed tree stands maintained SOC stocks and microbial activity comparable to those in reference forests, whereas monocultures experienced a decline. Differences in experimental scale, soil depth, stand age, and species dominance likely explain these inconsistencies.

In cocoa landscapes, farms often consist of mosaics of planted trees, remnant trees, and natural regeneration. Evidence shows that preserving remnant trees and leveraging natural regeneration can effectively re-establish multifunctional tree cover at scale, often outperforming mass tree planting (Kouassi et al., 2025; Konan et al., 2025). Shade tree species differ in effects on soil properties and crop competition; functional traits such as canopy architecture, phenology, and leaf nutrient content mediate impacts on cocoa productivity and soil processes (Addo-Danso et al., 2024; Isaac et al., 2024; Abdulai et al., 2025). Policy and restoration strategies must therefore consider species traits, placement, and farmer objectives to ensure ecological and socio-economic effectiveness.

Landscape and climate gradients further modulate tree diversity–soil relationships (Ding and Eldridge, 2021). Precipitation, temperature, and topography influence species pools and decomposition dynamics. Soil depth matters: studies that sample only the top 10 cm may miss significant root biomass and carbon inputs at depth (Fan et al., 2016; Cordeiro et al., 2020; Cusack and Turner, 2021; Krüger et al., 2024). Comprehensive assessments require sampling to 30 cm or deeper and integrating root distributions, microbial biomass, and carbon stabilization mechanisms (Losada et al., 2023; Fan et al., 2016).

Restoring functional tree cover in degraded cocoa landscapes can enhance aboveground carbon stocks and, under suitable species assemblages and management, improve soil health. Outcomes are contingent on species identity, stand age, and environmental context. Comparative studies across land-use systems, climate, geography, and soil depth are critical to inform ecologically effective and socio-economically feasible restoration strategies. This study addresses knowledge gaps on degraded cocoa landscapes in Côte d’Ivoire. Combining field inventories of tree diversity and functional composition with soil physicochemical and aboveground biomass carbon measurements across land-use, climate, and geographic gradients, we aimed to:

1. Quantify the relative influence of climate, geography, and land-use system on tree diversity and soil fertility;
2. Characterize tree diversity and soil property variation along climatic and geographic gradients;
3. Examine relationships among aboveground carbon, tree diversity, and soil properties across depths.

We tested three hypotheses:

(H1) Tree diversity is primarily determined by land-use systems, with fallows maintaining higher diversity than cocoa-based systems;
(H2) Climate and geography interact with land-use to influence soil fertility and tree diversity;
(H3) Species richness, rather than evenness and abundance, predicts soil fertility and aboveground carbon.

## 2 Materials and methods

### 2.1 Study area and sampling design

**Figure 1:**
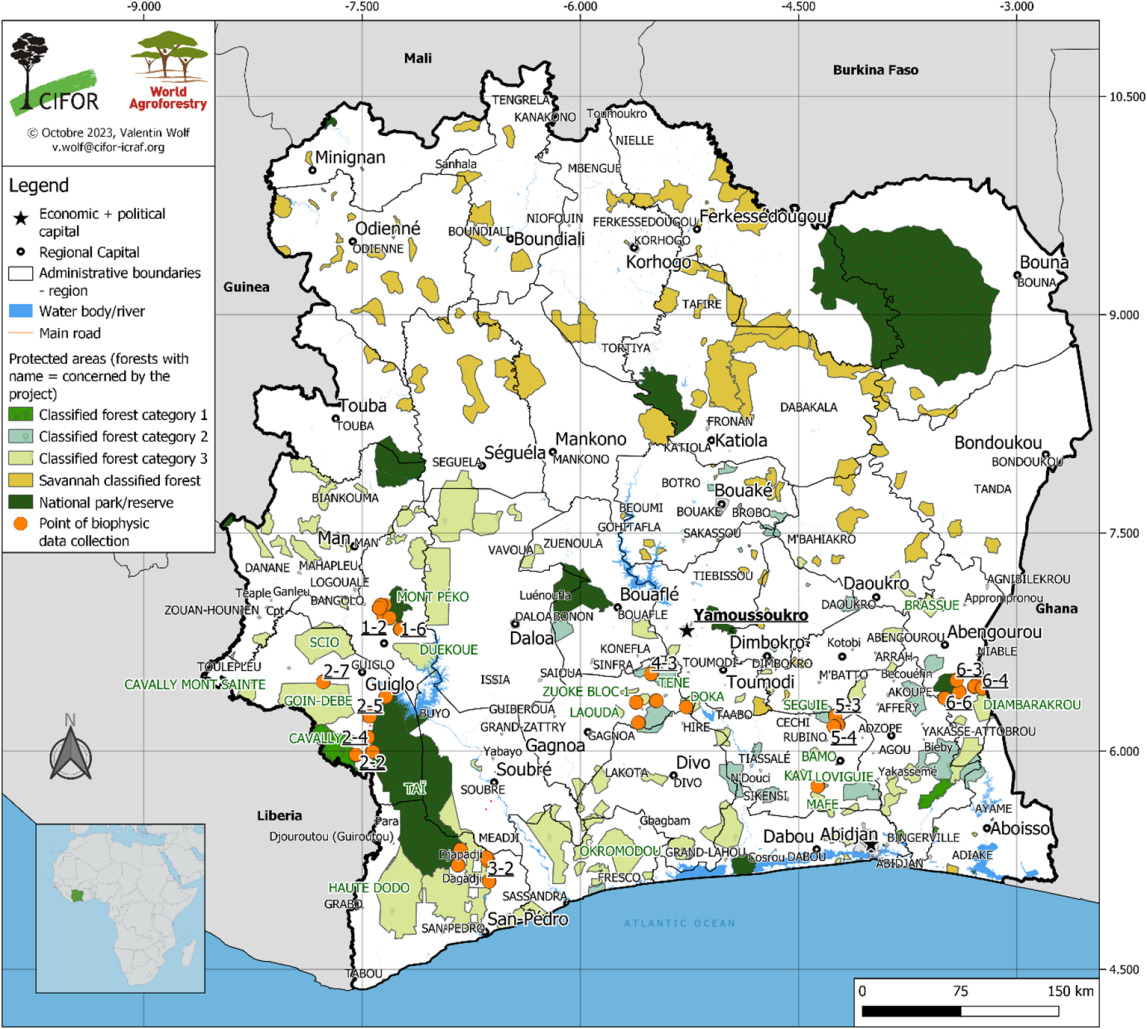
Plots and data collection clusters for studying the relationships between tree diversity, aboveground carbon, and soil fertility in cocoa-based land-use systems of Côte d’Ivoire (Cocoa farming area data source: Bureau National d’Etudes Techniques et Développement, Côte d’Ivoire, 2017).

Fieldwork was conducted across the major cocoa-producing zones of Côte d’Ivoire (**Figure 1**), spanning three distinct climatic regions: Climate Zone I Region I, Climate Zone I Region II, and Climate Zone VI Region II (**Table 1**; Goula et al., 2008). Sampling followed the Land Degradation Surveillance Framework (LDSF) (Vågen et al., 2012), a standardized, hierarchical approach for collecting biophysical data at the landscape scale that supports long-term monitoring of land degradation and rehabilitation efforts. A total of 32 sampling clusters were established, comprising 213 plots across the cocoa-growing forest zones (**Figure 1**). The region experiences a four-season tropical climate, with two rainy and two dry seasons, and peak rainfall typically in June and September (Goula et al., 2008). Annual precipitation ranges from approximately 1,150 mm in Zone I Region I to 1,650 mm in Zone VI Region II. Elevation varies from about 100 m a.s.l. in Oumé, Guiglo, and Duékoué to slightly lower in Abengourou, Djapadji, and Agboville (**Table 1**).

**Table 1:** Description of sampled sites used to assess variation in tree diversity and soil fertility indices in cocoa-based land-use systems in Côte d’Ivoire, and to evaluate the effects of tree diversity on soil stability and aboveground carbon sequestration (Sources: Dabin et al., 1960; Goula et al., 2008; Kassin et al., 2017)”.

Climate (humid tropical)		Site	Annual rainfall	Soils
Zone	Region			
I	I	Oumé	1150 mm	Complexes of slightly desaturated ferralitic soils and tropical brown soils; indurated reworked soils and eutrophic brown soils
	II	Abengourou	1200 mm	Ferralitic soils: Highly to slightly desaturated soils; gravelly soils
		Agboville	1200 mm	Feralitic soils; moderately desaturated; soils have deep or few gravel particles
	II	Djapadji	1200 mm	Gravelly soils
VI	II	Duékoué	1650 mm	Feralitic soils; moderately desaturated; soils have deep or few gravel particles
	II	Guiglo	1650 mm	Gravelly soils

Additional source information can be accessed in the References section).

Dominant soils are ferralitic, with desaturation levels differing by region: Guiglo and Djapadji exhibit highly desaturated soils, Abengourou ranges from highly to slightly desaturated, and Agboville and Duékoué show moderate desaturation. Oumé soils are characterized by slightly desaturated ferralitic and eutrophic brown soils (CNTIG, 2019; Dabin et al., 1960; Kassin et al., 2017). Soil texture and gravel content vary geographically, influencing soil fertility patterns.

### 2.2 Land-use systems

Cocoa plantations in Côte d’Ivoire are commonly established as monocultures (3 × 2.5 m spacing). However, farmers often retain or plant trees of socio-economic importance, providing fruits, seeds, leaves, and medicinal products. Following the ARS (African Regional Standard for Sustainable Cocoa) 1000–1 standard (ORAN-Organisation Africaine de Normalisation-, 2021), a cocoa farm was classified as an agroforestry system if it contained ≥ five non-cocoa trees per hectare. Agroforestry is defined as “a dynamic, ecologically based system integrating trees with crops and/or livestock to diversify and sustain production while enhancing social, economic, and environmental benefits” (Leakey, 1996). Land-use systems were therefore classified as cocoa monoculture and cocoa agroforestry, stratified by climate and geography to test H1.

### 2.3 Soil sampling and laboratory analysis

**Figure 2:**
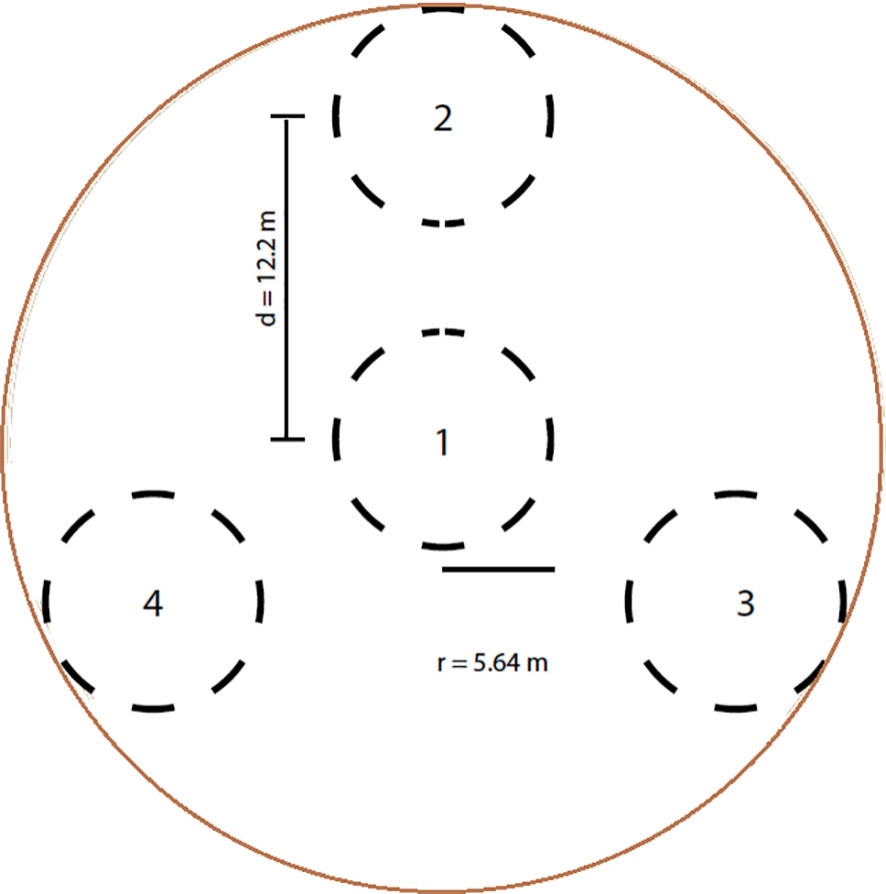
Sampled plots within clusters established to investigate the relationships among tree diversity, aboveground carbon biomass, and soil fertility in cocoa-based land-use systems of Côte d’Ivoire.

Each plot covered 1,000 m^2^ (radius = 17.84 m; **Figure 2**) and was subdivided into four subplots following LDSF standards. Composite soil samples (~500 g) were collected from each subplot at two depths: 0–20 cm and 20–50 cm, as most plant roots are concentrated within the upper 50 cm of the soil profile (Gao et al., 2016). These samples were used to test H1, which addressed depth-dependent effects on soil fertility and SOC. Sampling was conducted from 22 May to 20 June 2022, yielding 426 soil samples across the 213 plots. Samples were air-dried and sieved (2 mm). The coarse fraction (%) was calculated as:


Coarse fraction(%)=100×(weight of coarse fragments(g))/(total weight of air-dried soil(g))

Subsamples were ground and analyzed using mid-infrared (MIR) spectroscopy (Alpha MIR, Bruker Invenio-S FTIR). Each sample was scanned in duplicate, preprocessed with Savitzky–Golay smoothing (Savitzky and Golay, 1964) and mean-centered to reduce noise. Spectral data were projected onto a reference library, and calibration models were developed using Bidirectional Recurrent Neural Networks and Random Forest methods (Ateku and Chacha, 2021; Ateku, 2020). Calibration and validation sets included 5–95% of available values for each property, with outliers excluded to minimize bias. Predicted soil properties are displayed in **Table 2**.

**Table 2:** Predicted soil properties in an experiment investigating the relationships between tree biodiversity, soil fertility and aboveground carbon biomass in cocoa-based land-use systems and fallows across Côte d’Ivoire’s.

Property	Description	Unit
SOC	Organic Carbon content	% by weight
TN	Total Nitrogen content	% by weight
pH	Soil pH in water (soil: water ratio of 1:2 weight to volume basis)	–
m3.Al	Exchangeable aluminum concentration by Mehlich 3 extraction	mg/kg
m3.K	Potassium concentration by Mehlich 3 extraction	mg/kg
m3.Ca	Exchangeable calcium concentration by Mehlich 3 extraction	mg/kg
m3.Mg	Exchangeable Magnesium by Mehlich 3 extraction	mg/kg
m3.B	Boron concentration by Mehlich 3 extraction	mg/kg
m3.Fe	Iron concentration by Mehlich 3 extraction	mg/kg
CEC	Cation Exchange Capacity	cmolc/kg
ExAc	Exchangeable Acidity	cmolc/kg
PSI	Phosphorus Sorption Index	–

Soil structural stability was assessed using the Soil Structural Stability Index (SSSI) (Pieri, 1992):


SSSI=100×((SOC×1,724)/(clay(%)+silt(%))

### 2.4 Floristic data collection

Within each subplot, all trees ≥ 3 m in height were identified and counted following the LDSF protocol, which defines trees as woody vegetation equal or more than 3 m in height (Vågen and Winowiecki, 2023). Unidentified species were collected as leaf samples and later identified at the Centre National de Floristique, Université Félix Houphouët-Boigny (Abidjan). Nomenclature followed Lebrun and Stork (1997) and Aké Assi (2001, 2002), updated according to the Angiosperm Phylogeny Group IV (2016). These data supported H1 and H2, linking species composition and functional diversity to environmental gradients and soil fertility. The species richness was determined by counting all trees species recorded within each plot. Abundance was assessed based on the total number of individual trees of the different species identified in the plot.

The Shannon diversity index (H) and Evenness index (E) were calculated as follows:


$$\text{Shannon index}\left(\text{H}\right):\;\text{H}=-\sum \left(n_{i}/N\right)\;ln\;\left(n_{i}/N\right)$$

Evenness index (E) E=H/ln S

where n_i_ is the number of individuals of species i; N is the total number of individuals and S is the total number of species.

### 2.5 Estimation of aboveground biomass and carbon stocks

Tree height and circumference at breast height (1.30 m) were measured using a clinometer and tape. The diameter at breast height (DBH) was calculated as:


DBH=Circumference÷π

Aboveground biomass (AGB) was estimated using the allometric equation developed by Chave et al. (2014), expressed as:


$$\text{AGB}=\text{exp}(-2.024-0.896E+0.920\text{ln}\left(WD\right)+2.795\text{ln}\left(D\right)-0.046\left[\text{ln}\left(D\right){]}^{2}\right)$$

where *E* represents an environmental stress variable that increases with temperature seasonality, reflecting the duration of plant exposure to thermal stress (Chave et al., 2014); *WD* is the wood specific density (g cm^−3^); and *D* is the diameter at breast height (DBH, cm).

Species-specific values of *E* and *WD* were implemented using the BIOMASS package in R (Réjou-Méchain et al., 2017). AGB values were expressed in tonnes (t). Wood density data were obtained primarily from the Global Wood Density Database (Zanne, 2009). For species not listed in the database, a default value of 0.58 g cm^−3^ was applied (Reyes et al., 1992).

### 2.6 Hypothesis testing and data analysis

Analyses were conducted in SAS v9.04 (SAS Institute Inc, 2023) and R v4.5.1 (R Core Team, 2025). Prior to data analysis, the homogeneity of residual variances was tested graphically using proc plot in SAS, and the normality of residual variances was tested using proc univariate. Effects of climate, geography, and land-use system on tree diversity and soil fertility (H1 and H2) were assessed using a mixed-effects ANOVA (Montgomery, 2019):


$$Y_{ijk}=\mu +\lambda_{i}+\beta_{j\left(i\right)}+\delta_{k}+{\left(\delta \lambda \right)}_{ki}+{\left(\beta \delta \right)}_{jk\left(i\right)}+\epsilon_{ijk}$$

where $Y_{ijk}$ is the response, $\lambda_{i}$ is climate, $\beta_{j\left(i\right)}$ is site nested within climate, $\delta_{k}$ is land-use system, ${\left(\delta \lambda \right)}_{ki}$ is the climate × land-use interaction, ${\left(\beta \delta \right)}_{jk\left(i\right)}$ is land-use × site (nested in climate) interaction, and $\epsilon_{ijk}$ is random error. LSMeans were used for pairwise comparisons. Residual variance homogeneity and normality were verified prior to analysis.

Regression analyses explored relationships between geographic variables (elevation, latitude, longitude) and tree diversity indices (H1). Stepwise multiple regressions identified soil fertility and tree diversity predictors of aboveground carbon (H3) at 0.05 level. Redundancy analysis (RDA, vegan package in R; Oksanen et al., 2017) examined multivariate relationships between soil fertility and tree diversity across depths and land-use systems (H2 and H3).

## 3 Results

### 3.1 Effects of climate, geographic site, land-use system, and their interactions on tree diversity

Among the factors tested, land-use system had a highly significant effect (*P* < 0.0001) on tree species richness, abundance, evenness, and Shannon diversity indices (**Table 3**). The effects of land-use system on tree species richness, evenness and Shannon diversity were further modulated by geographic site nested within climate (**Table 3**). Geographic site nested within climate also showed a highly significant effect on tree species richness and Shannon diversity (**Table 3**). Additionally, both the climate × land-use system interaction (*P* = 0.001) and climate alone (*P* < 0.0001) had significant effects on species abundance.

**Table 3:** Fixed effects of tree diversity metrics in a nested climate–site–land-use design in cocoa-based systems of Côte d’Ivoire.

Source of variation	DF	Specific richness		Abundance		Evenness		Shannon index	
		*F* value	*P* value	F value	*P* value	F value	*P* value	*F* value	*P* value
Climate	2	1.20	0.3027	7.21	0.0010	1.06	0.3487	0.99	0.3746
Soil (Climate)	3	12.66	<0.0001	1.35	0.2606	2.06	0.1070	7.11	0.0001
Land-use system	2	58.92	<0.0001	15.94	<0.0001	86.92	<0.0001	85.84	<0.0001
Climate x Land-use system	3	1.75	0.1579	8.38	<0.0001	0.26	0.8559	0.12	0.9495
Land-use system x Geographic site (Climate)	6	4.76	0.0001	0.46	0.8378	2.35	0.0325	2.53	0.0221

Fallow systems exhibited the highest tree species richness index (7.2 ± 0.51), followed by agroforestry systems (4.1 ± 0.24). When considering the interaction between climate and land-use systems, fallows under the driest (Z1R1: 1150 mm annual rainfall) and slightly wetter (Z1R2: 1250 mm annual rainfall) climate zones showed the highest species richness indices (8.5 ± 0.86 and 7.4 ± 0.60, respectively; **Table 4**). Notably, fallows in Agboville, located within the Z1R2 climate zone, recorded the highest tree species richness index observed in the study (12.8 ± 1.15).

**Table 4:** Least-squares means comparisons of tree species richness across climate types nested within zones in degraded cocoa landscapes of Côte d’Ivoire.

Zone name	Climate*	Least-square means	Standard Error	Pr > |t|
Oume	Zone 1 Region1	6.36473430	0.50697454	<.0001
Abengourou	Zone 1 Region 2	4.28750000	0.53940548	<.0001
Agboville	Zone 1 Region 2	9.12727273	0.63882495	<.0001
San_Pedro	Zone 1 Region 2	3.38095238	0.62917533	<.0001
Duekoue	Zone 6 Region2	4.75757576	0.93898442	<.0001
Guiglo	Zone 6 Region 2	5.36363636	0.70087678	<.0001

*Zone 1 Region 1: Annual rainfall 1150 mm; Zone 1 Region 2: Annual rainfall 1200 mm; Zone 6 Region 2: Annual rainfall 1650 mm

Tree abundance was also greater in fallows (16 ± 1.80) than in agroforestry systems (8.2 ± 0.86). Among the sites, fallows in Oumé (Z1R1) exhibited the highest tree abundance (27.4 ± 3.03), followed by those in Agboville (17 ± 4.06) and Abengourou (17.5 ± 3.21), both located in the wetter Z1R2 zone. Overall, tree abundance was highest in the driest climate zone (17.5 ± 1.79), followed by the slightly wetter Z1R2 (9.7 ± 1.23), and lowest in the wettest zone, Z6R2 (9.1 ± 2.06).

Evenness did not differ significantly between fallows (0.98 ± 0.069) and agroforestry systems (0.96 ± 0.027). However, evenness was significantly lower (*P* < 0.0001) in monospecific cocoa systems (0.34 ± 0.047). Similarly, the Shannon diversity index was significantly higher in fallows (1.7 ± 0.05) than in agroforestry systems (1.2 ± 0.11; *P* < 0.0001). Among sites, Shannon indices for fallows in Agboville (1.9 ± 0.14) and Oumé (1.6 ± 0.14) were not significantly different (*P* = 0.057), yet they were the highest across all geographic zones nested within climate zones.

### 3.2 Effects of climate, geographic site, land-use system, and their interactions on soil metrics

Across soil depths, the geographic location of the site nested within climate had highly to very highly significant effects on nearly all measured soil parameters, with the exception of magnesium content at the 20–50 cm depth (**Tables 5A, B**). Climate, irrespective of soil depth, significantly influenced soil pH, aluminum, calcium, iron, magnesium, and cation exchange capacity (**Tables 5A, B**). At the 0–20 cm depth, climate also had significant to very highly significant effects on potassium (*P* = 0.0169) and the soil structural stability Index (*P* = 0.0008). At the 20–50 cm depth, climate had a marginally significant effect on phosphorus (*P* = 0.077) and a very highly significant effect on silt content (*P* < 0.0001).

**Table 5:** Mixed-Model ANOVA Results for Soil Health Indices: Comparison of three land-use systems (Fallow, cocoa agroforestry, and cocoa monocropping) across 213 Plots (1,000 m^2^ each) from six cocoa-producing sites in Côte d’Ivoire at 0–20 cm (A) and 20–50 cm (B) soil depths.

A: 0–20 cm soil depth																																	
Source of variation	Degrees of freedom	pH		Soil organic carbon		Total nitrogen		Aluminum		Boron		Calcium		Iron		Potassium		Magnesium		Exchangeable acidity		Phosphorus		Cation exchange capacity		Clay		Silt		Sand		Soil structural stability	
		F value	P	F value	P	F value	P	F value	P	F value	P	F value	P	F value	P	F value	P	F value	P	F value	P	F value	P	F value	P	F value	P	F value	P	F value	P	F value	P
Climate	2	17.29	<0.0001	1.99	0.1397	0.31	0.7312	4.97	0.0078	0.98	0.3768	4.80	0.0093	8.70	0.0002	4.17	0.0169	4.95	0.0080	2.37	0.0963	1.94	0.1471	7.44	0.0008	0.63	0.5360	2.33	0.099	1.26	0.2848	7.46	0.0008
Site (climate)	3	12.97	<0.0001	4.12	0.0073	5.92	0.0007	7.49	<0.0001	6.49	0.0003	6.37	0.0004	11.42	<0.0001	5.27	0.0016	4.65	0.0037	13.06	<0.0001	4.62	0.0038	6.92	0.0002	3.64	0.0137	10.36	<0.0001	4.52	0.0043	6.03	0.0006
Land-use system	2	0.78	0.4617	0.01	0.9895	0.04	0.9569	0.24	0.7862	0.33	0.7176	0.12	0.8830	0.55	0.5802	0.01	0.9920	0.05	0.9523	1.42	0.2433	0.35	0.7058	0.11	0.8961	0.01	0.9902	1.45	0.2379	0.14	0.8720	0.00	0.9990
Land-use system x climate	3	4.92	0.0025	2.98	0.0325	0.96	0.4144	1.24	0.2974	1.74	0.1697	3.87	0.0102	4.26	0.0061	4.45	0.0048	2.91	0.0358	0.69	0.5613	0.32	0.8080	3.93	0.0094	0.57	0.6322	0.85	0.4677	0.71	0.5497	2.97	0.0329
Land-use system x site (climate)	6	1	0.4270	1	0.4249	1.06	0.3867	2.01	0.0655	0.92	0.4843	0.40	0.8800	1.37	0.2281	0.29	0.941	1.11	0.3557	0.81	0.5636	1.23	0.2913	0.98	0.4375	1.89	0.0843	2.63	0.0179	2	0.0671	0.63	0.7088

B: 20–50 cm soil depth																																	
Source of variation	Degrees of freedom	pH		Soil organic carbon		Total nitrogen		Aluminum		Boron		Calcium		Iron		Potassium		Magnesium		Exchangeable acidity		Phosphorus		Cation exchange capacity		Clay		Silt		Sand		Soil structural stability	
		F value	P	F value	P	F value	P	F value	P	F value	P	F value	P	F value	P	F value	P	F value	P	F value	P	F value	P	F value	P	F value	P	F value	P	F value	P	F value	P
Climate	2	7.99	0.0005	0.01	0.9903	1.02	0.3617	5.68	0.0040	0.04	0.9585	3.36	0.0367	17.31	<0.0001	2.49	0.0863	3.34	0.0374	2.06	0.1303	4.99	0.0077	11.25	<0.0001	1.47	0.2330	11.62	<0.0001	1.99	0.1400	0.73	0.4818
Site (climate)	3	12.26	<0.0001	2.73	0.0448	5.88	0.0007	9.54	<0.0001	4.83	0.0029	9.50	<0.0001	13	<0.0001	6.40	0.0004	2.23	0.0857	11.34	<0.0001	6.33	0.0004	8.89	<0.0001	4.80	0.0030	5.23	0.0017	4.32	0.0056	4.73	0.0033
Land-use system	2	0.04	0.9647	0.52	0.5943	0.13	0.8805	0.43	0.6536	0.30	0.7419	0.11	0.8993	1.67	0.1906	0.67	0.5108	0.34	0.7103	0.29	0.7498	0.10	0.9077	0.11	0.8941	0.22	0.8011	2.03	0.1340	0.36	0.6995	0.77	0.4662
Land-use system x climate	3	1.11	0.3448	0.68	0.5640	0.62	0.6008	0.73	0.5359	0.87	0.4600	0.79	0.5036	4.15	0.0070	2.72	0.0462	0.35	0.7891	0.82	0.4848	0.77	0.5110	0.96	0.4143	0.82	0.4868	2.52	0.0595	0.56	0.6394	0.56	0.6430
Land-use system x site (climate)	6	1.28	0.2694	2.64	0.0175	1.84	0.0935	2.75	0.0136	2.24	0.0411	1.84	0.0933	1.36	0.2327	0.77	0.5928	1.24	0.2854	0.88	0.5098	1.38	0.2254	1.89	0.0845	1.91	0.0803	1.93	0.0770	2.13	0.0514	2.66	0.0108

The interaction between land-use system and climate significantly to very highly significantly affected soil pH, soil organic carbon, calcium, iron, potassium, magnesium, cation exchange capacity (CEC), and SSSI at the 0–20 cm depth (**Table 5A**). At the 20–50 cm depth, the land-use system × climate interaction had significant effects on soil iron (*P* = 0.0462) and highly significant effects on soil potassium content (*P* = 0.007). Additionally, the land-use system nested within climate significantly influenced SOC, aluminum, boron, and SSSI at the 20–50 cm depth (**Table 5B**).

At the 0–20 cm soil depth, soils in the driest climate zone (Z1R1) exhibited the highest pH values (6.7 ± 0.13), significantly higher than those in the wetter zones (Z1R2: 5.8 ± 0.09; Z6R2: 5.6 ± 0.15). A similar trend was observed at the 20–50 cm depth, where soils in the driest zone also had the highest pH (6.2 ± 0.11), compared to those in Z1R2 (5.7 ± 0.08) and Z6R2 (5.5 ± 0.13). However, at this depth, the difference between soils in Oumé (in the driest zone) and those in Abengourou (*P* = 0.1778) and Agboville (*P* = 0.4446) in wetter zones was not statistically significant.

At 20–50 cm depth, the highest soil organic carbon (SOC) contents were recorded in Duékoué (Z6R2: 0.8 ± 0.09%), Agboville (Z1R2: 0.8 ± 0.07%), and Abengourou (0.8 ± 0.006%). Across land-use systems at this depth, the highest SOC values were observed in fallows in Agboville (1.1 ± 0.15%), cocoa monocultures in Abengourou (0.9 ± 0.10%), fallows in Duékoué (0.9 ± 0.24%), agroforestry systems in Abengourou (0.8 ± 0.07%), monocultures in Duékoué (0.8 ± 0.15%), and agroforestry systems in Duékoué (0.8 ± 0.06%).

Total nitrogen (TN) content at 20–50 cm depth was highest in Duékoué (0.10 ± 0.01%) and was significantly higher than in soils from Oumé (*P* = 0.004), Djapadji (*P* = 0.0225), and Guiglo (*P* = 0.004). At the 0–20 cm soil depth, fallows in Abengourou and Duékoué, located in different climate zones, had the highest SOC values (1.1 ± 0.15% and 0.9 ± 0.24%, respectively). The highest TN concentrations were also recorded at this soil depth in Duékoué (0.13 ± 0.016%), Abengourou (0.11 ± 0.009%), and Agboville (0.10 ± 0.011%). In Duékoué, TN levels at 0–20 cm were significantly greater than those observed in Oumé (*P* = 0.0368), Djapadji (*P* = 0.0296), and Guiglo (*P* = 0.0071).

The soil structural stability index at 20–50 cm soil depth was significantly higher in Agboville (2.4 ± 0.18) than in the other sites, based on the climate × site interaction. At 0–20 cm soil depth, silt content was significantly greater in soils under wetter climates (Z1R2: 18.6 ± 0.41%; Z6R2: 17.5 ± 0.69%) compared to those in the drier zone (Z1R1: 16.4 ± 0.60%). At this same depth, soils from Abengourou exhibited significantly higher silt content (20.3 ± 0.63%) than those from Oumé (*P* < 0.0001), Djapadji (*P* = 0.0004), Duékoué (*P* = 0.0351), and Guiglo (*P* = 0.007). No significant difference was observed between silt content in Abengourou and Agboville (18.6 ± 0.75%) under the same climate (*P* = 0.0966). Additionally, no overall climate-related differences in silt content were detected at the 0–20 cm soil depth (*P* = 0.099). At 0–20 cm soil depth, the soil structural stability index was significantly higher in the driest climate zone (Z1R1: 4.0 ± 0.19) compared to wetter zones (Z6R2: 3.04 ± 0.22; Z1R2: 3.0 ± 0.13).

At 0–20 cm soil depth, aluminum concentrations were highest in soils under the Z6R2 climate zone (950.6 ± 48.29 mg/kg), compared to soils under Z1R2 (804 ± 28.75 mg/kg) and Z1R1 (714 ± 41.78 mg/kg). When examining the climate × geographical zone interaction, soils from Duékoué showed significantly higher aluminum levels (1134.8 ± 77.39 mg/kg) than those from Oumé (*P* < 0.0001), Abengourou (*P* = 0.0002), Agboville (*P* < 0.0001), Djapadji (*P* = 0.0139), and Guiglo (*P* = 0.0002).

At the same soil depth (0–20 cm), calcium concentrations were highest under the Z1R1 climate (1671.3 ± 210.57 mg/kg), compared to Z1R2 and Z6R2. The climate × site interaction showed that soils from Oumé had the highest calcium levels, significantly exceeding those of Djapadji (*P* = 0.0001) and Guiglo (*P* = 0.0003). For phosphorus, soils in the Z6R2 climate zone had the highest levels (86.1 ± 8.33 mg/kg), followed by Z1R2 (79.6 ± 4.96 mg/kg) and Z1R1 (61 ± 7.21 mg/kg). Djapadji soils recorded the highest phosphorus content (101 ± 8.95 mg/kg), which was significantly greater than in soils from Oumé (*P* = 0.0006), Abengourou (*P* = 0.0277), Agboville (*P* = 0.0033), and Guiglo (*P* = 0.0363), but not different from Duékoué (*P* = 0.9197).

At 20–50 cm soil depth, aluminum levels were again highest in the Z6R2 climate zone (1011.7 ± 53.77 mg/kg), followed by Z1R2 (826.4 ± 32.01 mg/kg) and Z1R1 (774.2 ± 46.53 mg/kg). Soils from Duékoué had the highest aluminum content (1230.1 ± 86.18 mg/kg), significantly exceeding levels in soils from Oumé, Abengourou, Agboville (all *P* < 0.0001), Djapadji (*P* = 0.0092), and Guiglo (*P* < 0.0001). At this same depth (20–50 cm), calcium levels were highest in soils from Agboville (1096.8 ± 149.29 mg/kg), significantly surpassing those from Djapadji and Guiglo (both *P* < 0.0001).

For potassium at 0–20 cm soil depth, Duékoué soils had the highest concentration (87.2 ± 13.28 mg/kg), significantly higher than Djapadji (*P* = 0.0048) and Guiglo (*P* = 0.0010). Regarding phosphorus at 0–20 cm, soils from the Z6R2 climate had the highest concentrations (124.5 ± 8.94 mg/kg), followed by Z1R2 (94.2 ± 5.33 mg/kg) and Z1R1 (83.8 ± 7.74 mg/kg). Duékoué again recorded the highest phosphorus content (152.1 ± 14.34 mg/kg), significantly higher than in soils from Oumé (*P* = 0.0007), Abengourou (*P* < 0.0001), Agboville (*P* < 0.0001), Djapadji (*P* = 0.05), and Guiglo (*P* = 0.0024).

At 20–50 cm soil depth, the highest CEC values across climatic zones were observed under climate Z1R1 (6.9 ± 9.63 cmolc/kg) and Z1R2 (6.7 ± 0.44 cmolc/kg), compared to soils under climate Z6R2 (3.9 ± 0.73 cmolc/kg). At the same depth (20–50 cm), the CEC of soils from Agboville (8.3 ± 0.80 cmolc/kg) was significantly higher than that of soils from Djapadji (*P* = 0.0036), Duékoué (P = 0.0089), and Guiglo (*P* < 0.0001). Over the 0–50 cm soil profile, soils under climates Z1R1 (10.9 ± 0.92 cmolc/kg) and Z1R2 (8.3 ± 0.63 cmolc/kg) exhibited higher CEC values than those under climate Z6R2. When nesting the geographic zone factor within climate, the highest CEC values were recorded in soils from Oumé (10.9 ± 0.92 cmolc/kg), Agboville (9.7 ± 1.55 cmolc/kg), and Abengourou (9 ± 0.75 cmolc/kg) at 0–50 cm soil depth.

### 3.3 Relationships between geographic variables and tree diversity metrics

Regression models assessing the relationships between altitude, latitude, longitude, and climate (predictor variables) and species richness, abundance, and the Shannon diversity index (response variables) were statistically significant (**Table 6**), with p-values of 0.0019, 0.0016, and 0.0003, respectively. In contrast, the model assessing the influence of these predictor variables on evenness was not significant (*P* = 0.0766). Among all predictors, climate emerged as the strongest determinant of species richness, abundance, and the Shannon index, with respective p-values of 0.030, 0.0127, and 0.0001. Altitude also significantly predicted species richness (*P* = 0.0298) and the Shannon index (*P* = 0.0106).

**Table 6:** Relationships between geographic variables and tree diversity metrics in degraded cocoa-based systems of Côte d’Ivoire.

		Species richness		Abundance		Evenness		Shannon index	
	Degrees of freedom	*F* value	*P* value	*F* value	*P* value	*F* value	*P* value	*F* value	*P* value
Altitude	1	4.79	0.0298	0.17	0.6801	0.50	0.4823	6.65	0.0106
Latitude	1	1.29	0.2575	0.01	0.9086	1.70	0.1941	1.65	0.2007
Longitude	1	0.44	0.5100	0.87	0.3518	0.38	0.5369	0.05	0.8257
Climate	2	5.99	0.0030	4.40	0.0127	3.81	0.0236	9.42	0.0001
Overall model	5	3.97	0.0019	4.05	0.0016	2.02	0.0760	4.93	0.0003

### 3.4 Relationships between aboveground carbon biomass and soil properties, and between aboveground carbon biomass and tree diversity

Stepwise regression (**Table 7**) identified aluminum content at 20–50 cm soil depth (F = 8.38; *P* = 0.0043), potassium at 0–20 cm soil depth (F = 9.78; *P* = 0.0021), boron at 20–50 cm soil depth (F = 4.00; *P* = 0.0471), and magnesium at 20–50 cm soil depth (F = 4.73; *P* = 0.0310) as significant soil predictors of aboveground carbon biomass (R^2^ = 0.1432; *P* < 0.0001). Among the tested tree diversity indices, only species richness significantly predicted aboveground carbon biomass (F = 30.49; *P* < 0.0001), with a model R^2^ of 0.1221 (*P* < 0.0001).

**Table 7:** Stepwise selection of soil fertility metrics that predict aboveground carbon biomass storage in degraded cocoa-based systems of Côte d’Ivoire.

Variable	Partial R-square	Model R-square	*F* value	*P* value
Aluminum (20-50cm soil depth)	0.0460	0.0460	8.38	0.0043
Potassium (0-20cm soil depth)	0.0511	0.0971	9.79	0.0021
Boron (20-50cm soil depth)	0.0205	0.1179	4.00	0.0471
Magnesium (20-50cm soil depth)	0.0238	0.1413	4.73	0.0310

### 3.5 Relationships between soil fertility metrics and tree diversity

Redundancy analysis revealed that species richness was the only tree diversity metric that tended to influence the soil fertility response variables (*P* = 0.0724; **Table 8**). However, the explanatory variables accounted for only 1.7% of the total variability in soil response variables. More specifically, species richness had a positive effect on soil structural stability index at both 0–20 cm (t = 3.14; *P* = 0.0019) and 20–50 cm soil depths (t = 2.29; *P* = 0.028), on soil magnesium content at 0–20 cm (t = 1.96; *P* = 0.0511), on soil organic carbon at 0–20 cm (t = 1.95; *P* = 0.0523), and on silt content at both 0–20 cm (t = 1.89; *P* = 0.0601) and 20–50 cm soil depths (t = 1.87; *P* = 0.0619).

**Table 8:** Relationships between soil properties and tree diversity metrics in degraded cocoa-based systems of Côte d’Ivoire.

Source of variation	Degrees of freedom	Variance	*F* value	*P* value
Species richness	1	0.2906	2.0491	0.07239
Abundance	1	0.1058	0.7462	0.55314
Evenness	1	0.0553	0.3901	0.89261
Shannon index	1	0.0506	0.3571	0.92591
Residual	208	29.4976		

## 4 Discussion

This study provides new empirical evidence that climate, geography, and land-use interactively regulate biodiversity, soil fertility, and carbon storage in degraded cocoa landscapes of Côte d’Ivoire. Among these factors, land-use system emerged as the most influential, confirming our first hypothesis that management intensity and vegetation structure strongly shape tree diversity. This finding aligns with growing evidence that diversified agroforestry systems can partially restore biodiversity and ecosystem functions within tropical agricultural mosaics (Montagnini and del Fierro, 2024; Martin et al., 2025; Mathieu et al., 2025). In such systems, vegetation complexity provides habitat heterogeneity, moderates microclimate, and sustains higher levels of ecological interactions compared to monocultures (Blaser et al., 2018).

The consistently higher tree richness and abundance in fallows compared to agroforestry and monoculture systems highlights the critical role of spontaneous regeneration in biodiversity recovery. Fallows act as reservoirs of native species and genetic resources, contributing to long-term resilience in cocoa landscapes (Chazdon and Brancalion, 2019; Araya et al., 2023). These findings corroborate previous reports that tropical agroforestry systems can retain 40–70% of forest tree diversity, depending on management intensity and proximity to forest remnants (Deheuvels et al., 2014; Haggar et al., 2019; Koelemeijer et al., 2021). The observed gradient—fallows > agroforests > monocultures—demonstrates how land-use intensification reduces the structural and compositional complexity that underpins ecological stability (Asigbaase et al., 2023).

In contrast, monoculture cocoa systems exhibited markedly lower diversity and evenness, consistent with previous studies (Niether et al., 2020; Boadi et al., 2024; Kamath et al., 2024). Ecological simplification in these systems diminishes pollination services, pest regulation, and nutrient cycling efficiency (Blaser et al., 2018). Our results reinforce the importance of biodiversity-friendly intensification, emphasizing the integration of native shade trees and conservation of remnant vegetation within cocoa farms (Asigbaase et al., 2023; Konan et al., 2025). Such strategies are central to reconciling productivity with conservation goals in tropical commodity landscapes (Tscharntke et al., 2023; Ahrens et al., 2025).

Tree diversity varied significantly among geographic sites nested within climatic zones, revealing that local biophysical and historical factors modulate regional climatic effects. Sites such as Agboville and Oumé—located in relatively drier zones—exhibited unexpectedly high species richness, likely due to the legacy of traditional agroforestry and longer fallow cycles. This supports evidence that landscape history and cultural management practices can override macroclimatic controls on biodiversity (Maney et al., 2022; Pascual et al., 2023; Maza-Villalobos et al., 2024). Interestingly, the highest abundance was observed in the driest zone (Z1R1), challenging the assumption that wetter climates inherently support denser vegetation (García-Callejas et al., 2017). This may reflect reduced land-use intensity and better regeneration potential in marginal areas less suitable for intensive cocoa production. These patterns emphasize that spatially explicit restoration strategies—integrating ecological gradients, land-use legacies, and socio-economic drivers—are essential for scaling up sustainable cocoa production in West Africa (Chazdon and Brancalion, 2019).

Soil fertility parameters were strongly influenced by both climatic and local factors, confirming our second hypothesis regarding interactive controls on soil properties. Significant climate × land-use interactions for soil organic carbon (SOC), pH, and base cations (Ca, Mg, K) indicate that environmental constraints mediate the outcomes of management interventions. Similar cross-scale patterns have been reported by Vicente et al. (2023) and Macedo et al. (2024), showing that tree-based systems enhance soil aggregation, nutrient retention, and cation exchange capacity through organic matter inputs. Higher SOC and total nitrogen in fallows and agroforests in Agboville and Duékoué confirm the soil-restorative function of tree cover, consistent with findings from West African parklands and cocoa agroforests (Bayala and Harmand, 2023). The diverse litter input from trees fosters microbial activity (Koné et al., 2021), which produces the humic binders essential for forming stable aggregates. The improvement in soil health indices we observe is therefore a direct consequence of the optimization of biogeochemical cycles induced by tree diversity.

Yet, relatively high SOC levels in some monocultures suggest that even simplified perennial systems can maintain moderate carbon stocks through litter inputs and reduced soil disturbance (Ngaba et al., 2024). In contrast, Koné and Yao (2021) demonstrated that mixed tree stands maintained SOC stocks and microbial activity comparable to those in reference forests, whereas monocultures. However, long-term resilience remains limited due to low functional diversity and reduced nutrient recycling capacity. Elevated pH and CEC values in drier zones further imply that lower leaching rates and slower organic matter decomposition can buffer soil fertility against degradation, an insight relevant for climate-resilient agroforestry interventions (Bayala and Harmand, 2023). These results confirm that macro-environmental factors modulate the effects of local management practices, aligning with global meta-analyses of soil–climate interactions in tropical agroecosystems (van der Sande et al., 2017).

Species richness emerged as a strong predictor of aboveground carbon biomass, validating our third hypothesis that compositional diversity outweighs functional diversity in determining carbon storage in degraded cocoa landscapes. This positive relationship aligns with previous findings showing that species-rich agroforestry systems often exceed monocultures in carbon storage potential (Mathieu et al., 2025; Dissanayaka et al., 2024). Mechanistically, high species richness enhances canopy stratification, light interception, and root complementarity, resulting in improved resource-use efficiency and biomass accumulation (Ledo et al., 2020).

Although low R^2^ values (0.12–0.14) suggest that additional factors—such as tree size structure, stand age, tree height structure, and management history—also influence aboveground carbon storage, the consistent positive link between aboveground carbon and soil nutrient availability (notably K, Mg, and B) highlights the interdependence between soil fertility and biomass productivity (van der Sande et al., 2017; Vicente et al., 2023). Accounting for stand structural attributes and management practices in future analyses could therefore help disentangle the relative contributions of tree species diversity and site carbon variation. Moreover, integrating nutrient management with tree diversification may provide synergistic benefits for enhancing both soil health and maximizing climate mitigation outcomes in degraded cocoa landscapes.

Although redundancy analysis explained only a modest proportion of variance, species richness significantly improved the soil structural stability index, confirming that diverse tree assemblages enhance soil aggregation and erosion resistance. This observation supports findings by Wen et al. (2021) and Ge et al. (2025), who reported that root diversity and organic inputs from mixed tree communities strengthen soil structure and water infiltration capacity. Similarly, Panchal et al. (2022) postulated that tree diversity fosters belowground carbon sequestration and hydrological regulation through litter quality variation and root–microbe interactions. The positive relationship we observed between tree species richness and soil structural stability is supported by underlying biological mechanisms. Indeed, Ntsoli et al. (2025) established that diversified systems promote a healthier and more functional microbial community; these microorganisms are essential for soil aggregate formation and thus for the physical stability we measured. Tree diversity thus acts as an optimizer of the soil carbon and nutrient cycles. The positive diversity–structure linkage observed here underscores the multifunctional role of biodiversity in agroecosystems: supporting soil fertility, water regulation, carbon storage, and resilience to climatic stressors (Tscharntke et al., 2023).

Collectively, these results highlight that biodiversity is not only an outcome of sustainable land use but also a driver of ecosystem multifunctionality in human-modified tropical landscapes. In cocoa systems under restoration, promoting diverse native species assemblages can accelerate recovery of soil fertility and carbon while buffering climate-related risks.

## 5 Conclusion

This study demonstrates that biodiversity, soil fertility, and carbon storage in cocoa landscapes are co-regulated by land-use, geography, and climate, with land-use management representing the most actionable lever for restoration. Increasing tree species richness within cocoa farms—particularly through native shade trees and spontaneous regeneration—emerges as a robust strategy to restore ecosystem functions and resilience in degraded West African landscapes.

Our findings reveal that species richness, rather than evenness and abundance, best predicts soil fertility and carbon accumulation, emphasizing the pivotal role of compositional diversity in ecosystem recovery. Macro-environmental gradients (climate and geography) modulate these relationships, suggesting that restoration efforts must be context-specific and spatially adaptive. Site-level interventions should thus be embedded within regional frameworks that consider climatic variability, landscape history, and socio-economic constraints.

From a policy and management perspective, promoting biodiversity-based restoration and farmer-managed natural regeneration (FMNR) within cocoa agroforests could significantly enhance ecosystem service delivery. Embedding these strategies within national climate and restoration policies—such as REDD+ programs, carbon markets, and payment for ecosystem services schemes—would provide concrete financial incentives for adoption. Strengthening local seed systems, conserving remnant vegetation, and incentivizing agroecological practices through results-based financing can further scale restoration outcomes. Future research should quantify belowground biomass and functional trait diversity contributions to carbon and nutrient cycling, and assess how restoration trajectories interact with climate variability to sustain productivity and resilience. Integrating long-term ecological monitoring with participatory approaches will be essential to co-design agroforestry systems that reconcile livelihood improvement with biodiversity conservation under changing climatic conditions.

## Data Availability

The datasets presented in this study can be found in online repositories. The names of the repository/repositories and accession number(s) can be found in the article/**Supplementary Material**.
