# Supplementary material for: Tree diversity and soil fertility interactions drive carbon storage in degraded cocoa landscapes of Côte d’Ivoire
Source: Front Plant Sci. 2025 Dec 16;16:1731574. doi: 10.3389/fpls.2025.1731574 (PMC12748207; doi:10.3389/fpls.2025.1731574)
Supplement: Supplementary file 4 [file DataSheet4.pdf]

Supplementary materials

Least-Square Means of tree diversity and soil fertility variables in the Ssudy ‘Tree diversity and soil fertility interactions drive carbon storage in degraded cocoa landscapes of Côte d’Ivoire

Dependent Variable: richness
richness

Least Squares Means

Zone_name	SAF_ID4	Climate	richness LSMEAN	Standard Error	Pr > |t|	LSMEAN Number
**Oume**	**AF**	**Z1R1**	4.1739130	0.4816927	<.0001	1
**Oume**	**Fallow**	**Z1R1**	8.5555556	0.7700390	<.0001	2
**Abengourou**	**AF**	**Z1R2**	3.4500000	0.5165578	<.0001	3
**Abengourou**	**Fallow**	**Z1R2**	5.1250000	0.8167497	<.0001	4
**Abengourou**	**Monocrop**	**Z1R2**	0.3636364	0.6965264	0.6022	5
**Agboville**	**AF**	**Z1R2**	5.4545455	0.4925186	<.0001	6
**Agboville**	**Fallow**	**Z1R2**	12.8000000	1.0331157	<.0001	7
**Agboville**	**Monocrop**	**Z1R2**	0.3333333	0.9431013	0.7241	8
**San_Pedro**	**AF**	**Z1R2**	2.4285714	0.6174047	0.0001	9
**San_Pedro**	**Fallow**	**Z1R2**	4.3333333	0.9431013	<.0001	10
**San_Pedro**	**Monocrop**	**Z1R2**	0.4285714	0.6174047	0.4884	11
**Duekoue**	**AF**	**Z6R2**	4.5151515	0.4021397	<.0001	12
**Duekoue**	**Fallow**	**Z6R2**	5.0000000	1.6334993	0.0025	13
**Duekoue**	**Monocrop**	**Z6R2**	0.6000000	1.0331157	0.5621	14
**Guiglo**	**AF**	**Z6R2**	4.2272727	0.4925186	<.0001	15
**Guiglo**	**Fallow**	**Z6R2**	6.5000000	1.1550584	<.0001	16
**Guiglo**	**Monocrop**	**Z6R2**	0.0000000	0.7700390	1.0000	17

Least Squares Means for effect Zone_*SAF_ID(Climat)
Pr > |t| for H0: LSMean(i)=LSMean(j)

Dependent Variable: richness

i/j	1	2	3	4	5	6	7	8	9	10	11	12
**1**		<.0001	0.3067	0.3171	<.0001	0.0645	<.0001	0.0004	0.0270	0.8805	<.0001	0.5872
**2**	<.0001		<.0001	0.0026	<.0001	0.0008	0.0012	<.0001	<.0001	0.0006	<.0001	<.0001
**3**	0.3067	<.0001		0.0846	0.0005	0.0055	<.0001	0.0042	0.2060	0.4124	0.0002	0.1053
**4**	0.3171	0.0026	0.0846		<.0001	0.7301	<.0001	0.0002	0.0091	0.5265	<.0001	0.5037
**5**	<.0001	<.0001	0.0005	<.0001		<.0001	<.0001	0.9794	0.0277	0.0009	0.9445	<.0001

Least Squares Means for effect Zone_*SAF_ID(Climat)<br>Pr > |t| for H0: LSMean(i)=LSMean(j)<br><br>Dependent Variable: richness												
i/j	1	2	3	4	5	6	7	8	9	10	11	12
6	0.0645	0.0008	0.0055	0.7301	<.0001		<.0001	<.0001	0.0002	0.2933	<.0001	0.1412
7	<.0001	0.0012	<.0001	<.0001	<.0001	<.0001		<.0001	<.0001	<.0001	<.0001	<.0001
8	0.0004	<.0001	0.0042	0.0002	0.9794	<.0001	<.0001		0.0646	0.0031	0.9328	<.0001
9	0.0270	<.0001	0.2060	0.0091	0.0277	0.0002	<.0001	0.0646		0.0927	0.0231	0.0051
10	0.8805	0.0006	0.4124	0.5265	0.0009	0.2933	<.0001	0.0031	0.0927		0.0007	0.8594
11	<.0001	<.0001	0.0002	<.0001	0.9445	<.0001	<.0001	0.9328	0.0231	0.0007		<.0001
12	0.5872	<.0001	0.1053	0.5037	<.0001	0.1412	<.0001	<.0001	0.0051	0.8594	<.0001	
13	0.6282	0.0504	0.3667	0.9455	0.0097	0.7902	<.0001	0.0142	0.1425	0.7241	0.0095	0.7735
14	0.0020	<.0001	0.0145	0.0007	0.8497	<.0001	<.0001	0.8490	0.1303	0.0082	0.8869	0.0005
15	0.9383	<.0001	0.2775	0.3477	<.0001	0.0796	<.0001	0.0003	0.0238	0.9207	<.0001	0.6512
16	0.0646	0.1403	0.0169	0.3323	<.0001	0.4061	<.0001	<.0001	0.0022	0.1478	<.0001	0.1062
17	<.0001	<.0001	0.0003	<.0001	0.7266	<.0001	<.0001	0.7845	0.0147	0.0005	0.6646	<.0001

Least Squares Means for effect Zone_*SAF_ID(Climat)<br>Pr > |t| for H0: LSMean(i)=LSMean(j)<br><br>Dependent Variable: richness					
i/j	13	14	15	16	17
1	0.6282	0.0020	0.9383	0.0646	<.0001
2	0.0504	<.0001	<.0001	0.1403	<.0001
3	0.3667	0.0145	0.2775	0.0169	0.0003
4	0.9455	0.0007	0.3477	0.3323	<.0001
5	0.0097	0.8497	<.0001	<.0001	0.7266
6	0.7902	<.0001	0.0796	0.4061	<.0001
7	<.0001	<.0001	<.0001	<.0001	<.0001
8	0.0142	0.8490	0.0003	<.0001	0.7845
9	0.1425	0.1303	0.0238	0.0022	0.0147
10	0.7241	0.0082	0.9207	0.1478	0.0005
11	0.0095	0.8869	<.0001	<.0001	0.6646
12	0.7735	0.0005	0.6512	0.1062	<.0001
13		0.0239	0.6511	0.4543	0.0062
14	0.0239		0.0018	0.0002	0.6420
15	0.6511	0.0018		0.0718	<.0001

Least Squares Means for effect Zone_*SAF_ID(Climat)<br>Pr > |t| for H0: LSMean(i)=LSMean(j)<br><br>Dependent Variable: richness					
i/j	13	14	15	16	17
16	0.4543	0.0002	0.0718		<.0001
17	0.0062	0.6420	<.0001	<.0001	

T Grouping for LS-Means of Zone_*SAF_ID(Climat)								
LS-means with the same letter are not significantly different.								
				richness LSMEAN	Zone_name	SAF_ID4	Climate	LSMEAN Number
		A		12.8000000	**Agboville**	**Fallow**	**Z1R2**	7
		B		8.5555556	**Oume**	**Fallow**	**Z1R1**	2
		B						
C		B		6.5000000	**Guiglo**	**Fallow**	**Z6R2**	16
C		B						
C		B		5.4545455	**Agboville**	**AF**	**Z1R2**	6
C		B						
C		B	D	5.1250000	**Abengourou**	**Fallow**	**Z1R2**	4
C		B	D					
C	E	B	D	5.0000000	**Duekoue**	**Fallow**	**Z6R2**	13
C	E		D					
C	E		D	4.5151515	**Duekoue**	**AF**	**Z6R2**	12
C	E		D					
C	E		D	4.3333333	**San_Pedro**	**Fallow**	**Z1R2**	10
C	E		D					
C	E		D	4.2272727	**Guiglo**	**AF**	**Z6R2**	15
C	E		D					
C	E		D	4.1739130	**Oume**	**AF**	**Z1R1**	1
	E		D					
	E		D	3.4500000	**Abengourou**	**AF**	**Z1R2**	3
	E							
	E	F		2.4285714	**San_Pedro**	**AF**	**Z1R2**	9
		F						
G		F		0.6000000	**Duekoue**	**Monocrop**	**Z6R2**	14
G		F						
G		F		0.4285714	**San_Pedro**	**Monocrop**	**Z1R2**	11
G		F						
G		F		0.3636364	**Abengourou**	**Monocrop**	**Z1R2**	5
G		F						
G		F		0.3333333	**Agboville**	**Monocrop**	**Z1R2**	8
G								

T Grouping for LS-Means of Zone_*SAF_ID(Climat)								
LS-means with the same letter are not significantly different.								
				richness LSMEAN	Zone_name	SAF_ID4	Climate	LSMEAN Number
G				0.0000000	Guiglo	Monocrop	Z6R2	17
The LINES display does not reflect all significant comparisons. The following additional pairs are significantly different: (2,6) (2,4) (12,9) (15,9) (1,9) (9,11) (9,5)								

Dependent Variable: abundance
abundance

Least Squares Means

Climate	SAF_ID4	abundance LSMEAN	Standard Error	Pr > |t|	LSMEAN Number
**Z1R1**	**AF**	7.6086957	1.6908878	<.0001	1
**Z1R1**	**Fallow**	27.4444444	2.7030710	<.0001	2
**Z1R2**	**AF**	6.1709957	1.1042405	<.0001	3
**Z1R2**	**Fallow**	13.1666667	1.8953649	<.0001	4
**Z1R2**	**Monocrop**	0.3751804	1.5504518	0.8090	5
**Z6R2**	**AF**	10.7803030	1.1159937	<.0001	6
**Z6R2**	**Fallow**	7.3750000	3.5113923	0.0370	7
**Z6R2**	**Monocrop**	0.3000000	2.2615515	0.8946	8

Least Squares Means for effect Climate*SAF_ID4
Pr > |t| for H0: LSMean(i)=LSMean(j)

Dependent Variable: abundance

i/j	1	2	3	4	5	6	7	8
**1**		<.0001	0.4774	0.0298	0.0019	0.1191	0.9522	0.0104
**2**	<.0001		<.0001	<.0001	<.0001	<.0001	<.0001	<.0001
**3**	0.4774	<.0001		0.0017	0.0026	0.0037	0.7439	0.0207
**4**	0.0298	<.0001	0.0017		<.0001	0.2793	0.1483	<.0001
**5**	0.0019	<.0001	0.0026	<.0001		<.0001	0.0697	0.9782
**6**	0.1191	<.0001	0.0037	0.2793	<.0001		0.3565	<.0001
**7**	0.9522	<.0001	0.7439	0.1483	0.0697	0.3565		0.0919
**8**	0.0104	<.0001	0.0207	<.0001	0.9782	<.0001	0.0919	

T Grouping for LS-Means of Climate*SAF_ID4

LS-means with the same letter are not significantly different.

			abundance LSMEAN	Climate	SAF_ID4	LSMEAN Number
	A		27.4444444	**Z1R1**	**Fallow**	2

T Grouping for LS-Means of Climate*SAF_ID4						
LS-means with the same letter are not significantly different.						
			abundance LSMEAN	Climate	SAF_ID4	LSMEAN Number
	B		13.1666667	Z1R2	Fallow	4
	B					
	B		10.7803030	Z6R2	AF	6
	B					
C	B		7.6086957	Z1R1	AF	1
C	B					
C	B	D	7.3750000	Z6R2	Fallow	7
C		D				
C		D	6.1709957	Z1R2	AF	3
		D				
		D	0.3751804	Z1R2	Monocrop	5
		D				
		D	0.3000000	Z6R2	Monocrop	8
The LINES display does not reflect all significant comparisons. The following additional pairs are significantly different: (4,1) (3,5) (3,8)						

Dependent Variable: Evenness
Evenness

Least Squares Means

Zone_name	SAF_ID4	Climate	Evenness LSMEAN	Standard Error	Pr > |t|	LSMEAN Number
Oume	AF	Z1R1	1.00000000	0.05386427	<.0001	1
Oume	Fallow	Z1R1	1.00000000	0.08610799	<.0001	2
Abengourou	AF	Z1R2	1.00000000	0.05776299	<.0001	3
Abengourou	Fallow	Z1R2	0.87500000	0.09133131	<.0001	4
Abengourou	Monocrop	Z1R2	0.36363636	0.07788761	<.0001	5
Agboville	AF	Z1R2	1.00000000	0.05507485	<.0001	6
Agboville	Fallow	Z1R2	1.00000000	0.11552599	<.0001	7
Agboville	Monocrop	Z1R2	0.33333333	0.10546032	0.0018	8

Zone_name	SAF_ID4	Climate	Evenness LSMEAN	Standard Error	Pr > |t|	LSMEAN Number
**San_Pedro**	**AF**	**Z1R2**	1.00000000	0.06903998	<.0001	9
**San_Pedro**	**Fallow**	**Z1R2**	1.00000000	0.10546032	<.0001	10
**San_Pedro**	**Monocrop**	**Z1R2**	0.42857143	0.06903998	<.0001	11
**Duekoue**	**AF**	**Z6R2**	0.93939394	0.04496843	<.0001	12
**Duekoue**	**Fallow**	**Z6R2**	1.00000000	0.18266263	<.0001	13
**Duekoue**	**Monocrop**	**Z6R2**	0.60000000	0.11552599	<.0001	14
**Guiglo**	**AF**	**Z6R2**	0.90909091	0.05507485	<.0001	15
**Guiglo**	**Fallow**	**Z6R2**	1.00000000	0.12916198	<.0001	16
**Guiglo**	**Monocrop**	**Z6R2**	0.00000000	0.08610799	1.0000	17

Least Squares Means for effect Zone_*SAF_ID(Climat)
Pr > |t| for H0: LSMean(i)=LSMean(j)

Dependent Variable: Evenness

i/j	1	2	3	4	5	6	7	8	9	10	11	12
**1**		1.0000	1.0000	0.2399	<.0001	1.0000	1.0000	<.0001	1.0000	1.0000	<.0001	0.3888
**2**	1.0000		1.0000	0.3206	<.0001	1.0000	1.0000	<.0001	1.0000	1.0000	<.0001	0.5334
**3**	1.0000	1.0000		0.2488	<.0001	1.0000	1.0000	<.0001	1.0000	1.0000	<.0001	0.4087
**4**	0.2399	0.3206	0.2488		<.0001	0.2426	0.3970	0.0001	0.2763	0.3714	0.0001	0.5278
**5**	<.0001	<.0001	<.0001	<.0001		<.0001	<.0001	0.8174	<.0001	<.0001	0.5334	<.0001
**6**	1.0000	1.0000	1.0000	0.2426	<.0001		1.0000	<.0001	1.0000	1.0000	<.0001	0.3950
**7**	1.0000	1.0000	1.0000	0.3970	<.0001	1.0000		<.0001	1.0000	1.0000	<.0001	0.6255
**8**	<.0001	<.0001	<.0001	0.0001	0.8174	<.0001	<.0001		<.0001	<.0001	0.4508	<.0001
**9**	1.0000	1.0000	1.0000	0.2763	<.0001	1.0000	1.0000	<.0001		1.0000	<.0001	0.4629
**10**	1.0000	1.0000	1.0000	0.3714	<.0001	1.0000	1.0000	<.0001	1.0000		<.0001	0.5977
**11**	<.0001	<.0001	<.0001	0.0001	0.5334	<.0001	<.0001	0.4508	<.0001	<.0001		<.0001
**12**	0.3888	0.5334	0.4087	0.5278	<.0001	0.3950	0.6255	<.0001	0.4629	0.5977	<.0001	
**13**	1.0000	1.0000	1.0000	0.5412	0.0016	1.0000	1.0000	0.0018	1.0000	1.0000	0.0038	0.7477
**14**	0.0020	0.0060	0.0022	0.0633	0.0914	0.0020	0.0152	0.0898	0.0033	0.0113	0.2043	0.0068
**15**	0.2394	0.3749	0.2561	0.7496	<.0001	0.2446	0.4783	<.0001	0.3046	0.4457	<.0001	0.6704
**16**	1.0000	1.0000	1.0000	0.4304	<.0001	1.0000	1.0000	<.0001	1.0000	1.0000	0.0001	0.6582
**17**	<.0001	<.0001	<.0001	<.0001	0.0020	<.0001	<.0001	0.0152	<.0001	<.0001	0.0001	<.0001

Least Squares Means for effect Zone_*SAF_ID(Climat)<br>Pr > |t| for H0: LSMean(i)=LSMean(j)<br><br>Dependent Variable: Evenness					
i/j	13	14	15	16	17
1	1.0000	0.0020	0.2394	1.0000	<.0001
2	1.0000	0.0060	0.3749	1.0000	<.0001
3	1.0000	0.0022	0.2561	1.0000	<.0001
4	0.5412	0.0633	0.7496	0.4304	<.0001
5	0.0016	0.0914	<.0001	<.0001	0.0020
6	1.0000	0.0020	0.2446	1.0000	<.0001
7	1.0000	0.0152	0.4783	1.0000	<.0001
8	0.0018	0.0898	<.0001	<.0001	0.0152
9	1.0000	0.0033	0.3046	1.0000	<.0001
10	1.0000	0.0113	0.4457	1.0000	<.0001
11	0.0038	0.2043	<.0001	0.0001	0.0001
12	0.7477	0.0068	0.6704	0.6582	<.0001
13		0.0657	0.6342	1.0000	<.0001
14	0.0657		0.0166	0.0220	<.0001
15	0.6342	0.0166		0.5181	<.0001
16	1.0000	0.0220	0.5181		<.0001
17	<.0001	<.0001	<.0001	<.0001	

T Grouping for LS-Means of Zone_*SAF_ID(Climat)						
LS-means with the same letter are not significantly different.						
		Evenness LSMEAN	Zone_name	SAF_ID4	Climate	LSMEAN Number
	A	1.00000000	**San_Pedro**	**AF**	**Z1R2**	9
	A					
	A	1.00000000	**Guiglo**	**Fallow**	**Z6R2**	16
	A					
	A	1.00000000	**Oume**	**AF**	**Z1R1**	1
	A					
	A	1.00000000	**San_Pedro**	**Fallow**	**Z1R2**	10
	A					
	A	1.00000000	**Agboville**	**Fallow**	**Z1R2**	7
	A					
	A	1.00000000	**Agboville**	**AF**	**Z1R2**	6
	A					
B	A	1.00000000	**Duekoue**	**Fallow**	**Z6R2**	13
B	A					
B	A	1.00000000	**Abengourou**	**AF**	**Z1R2**	3
B	A					
B	A	1.00000000	**Oume**	**Fallow**	**Z1R1**	2
B	A					
B	A	0.93939394	**Duekoue**	**AF**	**Z6R2**	12
B	A					
B	A	0.90909091	**Guiglo**	**AF**	**Z6R2**	15
B	A					
B	A	0.87500000	**Abengourou**	**Fallow**	**Z1R2**	4
B						
B	C	0.60000000	**Duekoue**	**Monocrop**	**Z6R2**	14
	C					
	C	0.42857143	**San_Pedro**	**Monocrop**	**Z1R2**	11
	C					
	C	0.36363636	**Abengourou**	**Monocrop**	**Z1R2**	5
	C					
	C	0.33333333	**Agboville**	**Monocrop**	**Z1R2**	8

T Grouping for LS-Means of Zone_*SAF_ID(Climat)						
LS-means with the same letter are not significantly different.						
		Evenness LSMEAN	Zone_name	SAF_ID4	Climate	LSMEAN Number
	D	0.00000000	Guiglo	Monocrop	Z6R2	17
The LINES display does not reflect all significant comparisons. The following additional pairs are significantly different: (3,14) (2,14) (12,14) (15,14)						

Dependent Variable: Shannon
Shannon

Least Squares Means

Zone_name	SAF_ID4	Climate	Shannon LSMEAN	Standard Error	Pr > |t|	LSMEAN Number
Oume	AF	Z1R1	1.26086957	0.10829473	<.0001	1
Oume	Fallow	Z1R1	1.88888889	0.17312110	<.0001	2
Abengourou	AF	Z1R2	0.95000000	0.11613316	<.0001	3
Abengourou	Fallow	Z1R2	1.00000000	0.18362265	<.0001	4
Abengourou	Monocrop	Z1R2	0.00000000	0.15659392	1.0000	5
Agboville	AF	Z1R2	1.45454545	0.11072863	<.0001	6
Agboville	Fallow	Z1R2	2.40000000	0.23226633	<.0001	7
Agboville	Monocrop	Z1R2	0.00000000	0.21202918	1.0000	8
San_Pedro	AF	Z1R2	0.78571429	0.13880568	<.0001	9
San_Pedro	Fallow	Z1R2	1.50000000	0.21202918	<.0001	10
San_Pedro	Monocrop	Z1R2	-0.00000000	0.13880568	1.0000	11
Duekoue	AF	Z6R2	1.15151515	0.09040954	<.0001	12
Duekoue	Fallow	Z6R2	1.50000000	0.36724531	<.0001	13
Duekoue	Monocrop	Z6R2	-0.00000000	0.23226633	1.0000	14
Guiglo	AF	Z6R2	1.13636364	0.11072863	<.0001	15
Guiglo	Fallow	Z6R2	1.75000000	0.25968165	<.0001	16
Guiglo	Monocrop	Z6R2	0.00000000	0.17312110	1.0000	17

Least Squares Means for effect Zone_*SAF_ID(Climat) Pr > |t| for H0: LSMean(i)=LSMean(j) Dependent Variable: Shannon												
i/j	1	2	3	4	5	6	7	8	9	10	11	12
1		0.0024	0.0517	0.2225	<.0001	0.2126	<.0001	<.0001	0.0076	0.3164	<.0001	0.4392
2	0.0024		<.0001	0.0005	<.0001	0.0358	0.0792	<.0001	<.0001	0.1570	<.0001	0.0002
3	0.0517	<.0001		0.8182	<.0001	0.0019	<.0001	0.0001	0.3651	0.0240	<.0001	0.1725
4	0.2225	0.0005	0.8182		<.0001	0.0353	<.0001	0.0005	0.3530	0.0762	<.0001	0.4600
5	<.0001	<.0001	<.0001	<.0001		<.0001	<.0001	1.0000	0.0002	<.0001	1.0000	<.0001
6	0.2126	0.0358	0.0019	0.0353	<.0001		0.0003	<.0001	0.0002	0.8495	<.0001	0.0353
7	<.0001	0.0792	<.0001	<.0001	<.0001	0.0003		<.0001	<.0001	0.0047	<.0001	<.0001
8	<.0001	<.0001	0.0001	0.0005	1.0000	<.0001	<.0001		0.0022	<.0001	1.0000	<.0001
9	0.0076	<.0001	0.3651	0.3530	0.0002	0.0002	<.0001	0.0022		0.0053	<.0001	0.0284
10	0.3164	0.1570	0.0240	0.0762	<.0001	0.8495	0.0047	<.0001	0.0053		<.0001	0.1322
11	<.0001	<.0001	<.0001	<.0001	1.0000	<.0001	<.0001	1.0000	<.0001	<.0001		<.0001
12	0.4392	0.0002	0.1725	0.4600	<.0001	0.0353	<.0001	<.0001	0.0284	0.1322	<.0001	
13	0.5330	0.3393	0.1549	0.2248	0.0002	0.9058	0.0396	0.0005	0.0704	1.0000	0.0002	0.3580
14	<.0001	<.0001	0.0003	0.0009	1.0000	<.0001	<.0001	1.0000	0.0041	<.0001	1.0000	<.0001
15	0.4224	0.0003	0.2469	0.5256	<.0001	0.0435	<.0001	<.0001	0.0497	0.1301	<.0001	0.9157
16	0.0837	0.6568	0.0054	0.0194	<.0001	0.2966	0.0636	<.0001	0.0012	0.4567	<.0001	0.0307
17	<.0001	<.0001	<.0001	0.0001	1.0000	<.0001	<.0001	1.0000	0.0005	<.0001	1.0000	<.0001

Least Squares Means for effect Zone_*SAF_ID(Climat) Pr > |t| for H0: LSMean(i)=LSMean(j) Dependent Variable: Shannon					
i/j	13	14	15	16	17
1	0.5330	<.0001	0.4224	0.0837	<.0001
2	0.3393	<.0001	0.0003	0.6568	<.0001
3	0.1549	0.0003	0.2469	0.0054	<.0001
4	0.2248	0.0009	0.5256	0.0194	0.0001
5	0.0002	1.0000	<.0001	<.0001	1.0000
6	0.9058	<.0001	0.0435	0.2966	<.0001
7	0.0396	<.0001	<.0001	0.0636	<.0001
8	0.0005	1.0000	<.0001	<.0001	1.0000
9	0.0704	0.0041	0.0497	0.0012	0.0005
10	1.0000	<.0001	0.1301	0.4567	<.0001

Least Squares Means for effect Zone_*SAF_ID(Climat) Pr > |t| for H0: LSMean(i)=LSMean(j) Dependent Variable: Shannon					
i/j	13	14	15	16	17
11	0.0002	1.0000	<.0001	<.0001	1.0000
12	0.3580	<.0001	0.9157	0.0307	<.0001
13		0.0007	0.3443	0.5790	0.0003
14	0.0007		<.0001	<.0001	1.0000
15	0.3443	<.0001		0.0309	<.0001
16	0.5790	<.0001	0.0309		<.0001
17	0.0003	1.0000	<.0001	<.0001	

T Grouping for LS-Means of Zone_*SAF_ID(Climat)							
LS-means with the same letter are not significantly different.							
			Shannon LSMEAN	Zone_name	SAF_ID4	Climate	LSMEAN Number
	A		2.40000000	**Agboville**	**Fallow**	**Z1R2**	7
	A						
B	A		1.88888889	**Oume**	**Fallow**	**Z1R1**	2
B	A						
B	A	C	1.75000000	**Guiglo**	**Fallow**	**Z6R2**	16
B		C					
B	D	C	1.50000000	**Duekoue**	**Fallow**	**Z6R2**	13
B	D	C					
B	D	C	1.50000000	**San_Pedro**	**Fallow**	**Z1R2**	10
	D	C					
	D	C	1.45454545	**Agboville**	**AF**	**Z1R2**	6
	D	C					
	D	C	1.26086957	**Oume**	**AF**	**Z1R1**	1
	D						
	D		1.15151515	**Duekoue**	**AF**	**Z6R2**	12
	D						
	D		1.13636364	**Guiglo**	**AF**	**Z6R2**	15
	D						
	D		1.00000000	**Abengourou**	**Fallow**	**Z1R2**	4
	D						
	D		0.95000000	**Abengourou**	**AF**	**Z1R2**	3
	D						
	D		0.78571429	**San_Pedro**	**AF**	**Z1R2**	9
	E		0.00000000	**Guiglo**	**Monocrop**	**Z6R2**	17
	E						
	E		0.00000000	**Abengourou**	**Monocrop**	**Z1R2**	5
	E						
	E		0.00000000	**Agboville**	**Monocrop**	**Z1R2**	8
	E						
	E		-0.00000000	**San_Pedro**	**Monocrop**	**Z1R2**	11
	E						
	E		-0.00000000	**Duekoue**	**Monocrop**	**Z6R2**	14

T Grouping for LS-Means of Zone_*SAF_ID(Climat)					
LS-means with the same letter are not significantly different.					
	Shannon LSMEAN	Zone_name	SAF_ID4	Climate	LSMEAN Number
The LINES display does not reflect all significant comparisons. The following additional pairs are significantly different: (10,3) (10,9) (6,12) (6,15) (6,4) (6,3) (6,9) (1,9) (12,9) (15,9)					

Dependent Variable: pH
pH

Least Squares Means

Zone_name	Climate	pH LSMEAN	Standard Error	Pr > |t|	LSMEAN Number
**Oume**	**Z1R1**	Non-est	.	.	1
**Abengourou**	**Z1R2**	5.87606061	0.09490232	<.0001	2
**Agboville**	**Z1R2**	5.95807071	0.11814700	<.0001	3
**San_Pedro**	**Z1R2**	5.23746032	0.10238993	<.0001	4
**Duekoue**	**Z6R2**	5.79778788	0.15727575	<.0001	5
**Guiglo**	**Z6R2**	5.23265152	0.11734797	<.0001	6

Least Squares Means for effect Zone_name(Climate)<br>Pr > |t| for H0: LSMean(i)=LSMean(j)<br><br>Dependent Variable: pH						
**i/j**	**1**	**2**	**3**	**4**	**5**	**6**
**1**		.	.	.	.	.
**2**	.		0.5890	<.0001	0.6705	<.0001
**3**	.	0.5890		<.0001	0.4162	<.0001
**4**	.	<.0001	<.0001		0.0032	0.9754
**5**	.	0.6705	0.4162	0.0032		0.0044
**6**	.	<.0001	<.0001	0.9754	0.0044	

Dependent Variable: SOC
SOC

Least Squares Means

Zone_name	SAF_ID4	Climate	SOC LSMEAN	Standard Error	Pr > |t|	LSMEAN Number
**Oume**	**AF**	**Z1R1**	0.59782609	0.07260978	<.0001	1
**Oume**	**Fallow**	**Z1R1**	0.81666667	0.11607475	<.0001	2
**Abengourou**	**AF**	**Z1R2**	0.83150000	0.07786531	<.0001	3
**Abengourou**	**Fallow**	**Z1R2**	0.59000000	0.12311586	<.0001	4
**Abengourou**	**Monocrop**	**Z1R2**	0.88181818	0.10499356	<.0001	5
**Agboville**	**AF**	**Z1R2**	0.60818182	0.07424166	<.0001	6
**Agboville**	**Fallow**	**Z1R2**	1.09400000	0.15573062	<.0001	7
**Agboville**	**Monocrop**	**Z1R2**	0.67000000	0.14216195	<.0001	8
**San_Pedro**	**AF**	**Z1R2**	0.67857143	0.09306685	<.0001	9
**San_Pedro**	**Fallow**	**Z1R2**	0.71000000	0.14216195	<.0001	10
**San_Pedro**	**Monocrop**	**Z1R2**	0.43642857	0.09306685	<.0001	11
**Duekoue**	**AF**	**Z6R2**	0.83393939	0.06061806	<.0001	12
**Duekoue**	**Fallow**	**Z6R2**	0.90000000	0.24623173	0.0003	13
**Duekoue**	**Monocrop**	**Z6R2**	0.77000000	0.15573062	<.0001	14
**Guiglo**	**AF**	**Z6R2**	0.70500000	0.07424166	<.0001	15
**Guiglo**	**Fallow**	**Z6R2**	0.48250000	0.17411213	0.0061	16
**Guiglo**	**Monocrop**	**Z6R2**	0.60444444	0.11607475	<.0001	17

Least Squares Means for effect Zone_*SAF_ID(Climat)
Pr > |t| for H0: LSMean(i)=LSMean(j)

Dependent Variable: SOC

i/j	1	2	3	4	5	6	7	8	9	10	11	12
**1**		0.1116	0.0294	0.9564	0.0272	0.9207	0.0043	0.6517	0.4948	0.4831	0.1731	0.0134
**2**	0.1116		0.9156	0.1819	0.6777	0.1319	0.1549	0.4252	0.3544	0.5618	0.0114	0.8952
**3**	0.0294	0.9156		0.0990	0.7007	0.0392	0.1333	0.3203	0.2091	0.4544	0.0013	0.9803
**4**	0.9564	0.1819	0.0990		0.0728	0.8995	0.0119	0.6710	0.5667	0.5242	0.3209	0.0770
**5**	0.0272	0.6777	0.7007	0.0728		0.0346	0.2600	0.2322	0.1490	0.3321	0.0017	0.6933
**6**	0.9207	0.1319	0.0392	0.8995	0.0346		0.0054	0.7003	0.5550	0.5263	0.1507	0.0195
**7**	0.0043	0.1549	0.1333	0.0119	0.2600	0.0054		0.0457	0.0231	0.0701	0.0004	0.1213
**8**	0.6517	0.4252	0.3203	0.6710	0.2322	0.7003	0.0457		0.9598	0.8425	0.1708	0.2901
**9**	0.4948	0.3544	0.2091	0.5667	0.1490	0.5550	0.0231	0.9598		0.8534	0.0673	0.1634
**10**	0.4831	0.5618	0.4544	0.5242	0.3321	0.5263	0.0701	0.8425	0.8534		0.1090	0.4235
**11**	0.1731	0.0114	0.0013	0.3209	0.0017	0.1507	0.0004	0.1708	0.0673	0.1090		0.0004
**12**	0.0134	0.8952	0.9803	0.0770	0.6933	0.0195	0.1213	0.2901	0.1634	0.4235	0.0004	
**13**	0.2406	0.7598	0.7911	0.2615	0.9459	0.2579	0.5063	0.4195	0.4013	0.5048	0.0798	0.7947

Least Squares Means for effect Zone_*SAF_ID(Climat) Pr > |t| for H0: LSMean(i)=LSMean(j) Dependent Variable: SOC												
**i/j**	**1**	**2**	**3**	**4**	**5**	**6**	**7**	**8**	**9**	**10**	**11**	**12**
**14**	0.3176	0.8104	0.7243	0.3657	0.5523	0.3494	0.1429	0.6359	0.6149	0.7763	0.0675	0.7024
**15**	0.3033	0.4187	0.2411	0.4247	0.1707	0.3576	0.0253	0.8275	0.8246	0.9752	0.0252	0.1801
**16**	0.5417	0.1119	0.0688	0.6147	0.0509	0.5075	0.0095	0.4052	0.3219	0.3127	0.8157	0.0581
**17**	0.9615	0.1976	0.1059	0.9321	0.0779	0.9784	0.0125	0.7213	0.6189	0.5659	0.2601	0.0812

Least Squares Means for effect Zone_*SAF_ID(Climat) Pr > |t| for H0: LSMean(i)=LSMean(j) Dependent Variable: SOC					
**i/j**	**13**	**14**	**15**	**16**	**17**
**1**	0.2406	0.3176	0.3033	0.5417	0.9615
**2**	0.7598	0.8104	0.4187	0.1119	0.1976
**3**	0.7911	0.7243	0.2411	0.0688	0.1059
**4**	0.2615	0.3657	0.4247	0.6147	0.9321
**5**	0.9459	0.5523	0.1707	0.0509	0.0779
**6**	0.2579	0.3494	0.3576	0.5075	0.9784
**7**	0.5063	0.1429	0.0253	0.0095	0.0125
**8**	0.4195	0.6359	0.8275	0.4052	0.7213
**9**	0.4013	0.6149	0.8246	0.3219	0.6189
**10**	0.5048	0.7763	0.9752	0.3127	0.5659
**11**	0.0798	0.0675	0.0252	0.8157	0.2601
**12**	0.7947	0.7024	0.1801	0.0581	0.0812
**13**		0.6559	0.4492	0.1678	0.2789
**14**	0.6559		0.7068	0.2199	0.3951
**15**	0.4492	0.7068		0.2412	0.4664
**16**	0.1678	0.2199	0.2412		0.5607
**17**	0.2789	0.3951	0.4664	0.5607	

T Grouping for LS-Means of Zone_*SAF_ID(Climat)						
LS-means with the same letter are not significantly different.						
		SOC LSMEAN	Zone_name	SAF_ID4	Climate	LSMEAN Number
	A	1.09400000	**Agboville**	**Fallow**	**Z1R2**	7
	A					
B	A	0.90000000	**Duekoue**	**Fallow**	**Z6R2**	13
B	A					
B	A	0.88181818	**Abengourou**	**Monocrop**	**Z1R2**	5
B	A					
B	A	0.83393939	**Duekoue**	**AF**	**Z6R2**	12
B	A					
B	A	0.83150000	**Abengourou**	**AF**	**Z1R2**	3
B	A					
B	A	0.81666667	**Oume**	**Fallow**	**Z1R1**	2
B	A					
B	A	0.77000000	**Duekoue**	**Monocrop**	**Z6R2**	14
B	A					
B	A	0.71000000	**San_Pedro**	**Fallow**	**Z1R2**	10
B						
B		0.70500000	**Guiglo**	**AF**	**Z6R2**	15
B						
B		0.67857143	**San_Pedro**	**AF**	**Z1R2**	9
B						
B		0.67000000	**Agboville**	**Monocrop**	**Z1R2**	8
B						
B		0.60818182	**Agboville**	**AF**	**Z1R2**	6
B						
B		0.60444444	**Guiglo**	**Monocrop**	**Z6R2**	17
B						
B		0.59782609	**Oume**	**AF**	**Z1R1**	1
B						
B		0.59000000	**Abengourou**	**Fallow**	**Z1R2**	4
B						
B		0.48250000	**Guiglo**	**Fallow**	**Z6R2**	16
B						
B		0.43642857	**San_Pedro**	**Monocrop**	**Z1R2**	11

T Grouping for LS-Means of Zone_*SAF_ID(Climat)					
LS-means with the same letter are not significantly different.					
	SOC LSMEAN	Zone_name	SAF_ID4	Climate	LSMEAN Number
The LINES display does not reflect all significant comparisons. The following additional pairs are significantly different: (5,6) (5,1) (5,11) (12,6) (12,1) (12,11) (3,6) (3,1) (3,11) (2,11) (15,11)					

Dependent Variable: SOM
SOM

Least Squares Means

Zone_name	SAF_ID4	Climate	SOM LSMEAN	Standard Error	Pr > |t|	LSMEAN Number
Oume	AF	Z1R1	1.03065217	0.12517925	<.0001	1
Oume	Fallow	Z1R1	1.40793333	0.20011287	<.0001	2
Abengourou	AF	Z1R2	1.43350600	0.13423979	<.0001	3
Abengourou	Fallow	Z1R2	1.01716000	0.21225175	<.0001	4
Abengourou	Monocrop	Z1R2	1.52025455	0.18100890	<.0001	5
Agboville	AF	Z1R2	1.04850545	0.12799262	<.0001	6
Agboville	Fallow	Z1R2	1.88605600	0.26847959	<.0001	7
Agboville	Monocrop	Z1R2	1.15508000	0.24508721	<.0001	8
San_Pedro	AF	Z1R2	1.16985714	0.16044724	<.0001	9
San_Pedro	Fallow	Z1R2	1.22404000	0.24508721	<.0001	10
San_Pedro	Monocrop	Z1R2	0.75240286	0.16044724	<.0001	11
Duekoue	AF	Z6R2	1.43771152	0.10450554	<.0001	12
Duekoue	Fallow	Z6R2	1.55160000	0.42450350	0.0003	13
Duekoue	Monocrop	Z6R2	1.32748000	0.26847959	<.0001	14
Guiglo	AF	Z6R2	1.21542000	0.12799262	<.0001	15
Guiglo	Fallow	Z6R2	0.83183000	0.30016930	0.0061	16
Guiglo	Monocrop	Z6R2	1.04206222	0.20011287	<.0001	17

Least Squares Means for effect Zone_*SAF_ID(Climat)<br>Pr > |t| for H0: LSMean(i)=LSMean(j)<br><br>Dependent Variable: SOM												
i/j	1	2	3	4	5	6	7	8	9	10	11	12
1		0.1116	0.0294	0.9564	0.0272	0.9207	0.0043	0.6517	0.4948	0.4831	0.1731	0.0134

Least Squares Means for effect Zone_*SAF_ID(Climat) Pr > |t| for H0: LSMean(i)=LSMean(j) Dependent Variable: SOM												
**i/j**	**1**	**2**	**3**	**4**	**5**	**6**	**7**	**8**	**9**	**10**	**11**	**12**
**2**	0.1116		0.9156	0.1819	0.6777	0.1319	0.1549	0.4252	0.3544	0.5618	0.0114	0.8952
**3**	0.0294	0.9156		0.0990	0.7007	0.0392	0.1333	0.3203	0.2091	0.4544	0.0013	0.9803
**4**	0.9564	0.1819	0.0990		0.0728	0.8995	0.0119	0.6710	0.5667	0.5242	0.3209	0.0770
**5**	0.0272	0.6777	0.7007	0.0728		0.0346	0.2600	0.2322	0.1490	0.3321	0.0017	0.6933
**6**	0.9207	0.1319	0.0392	0.8995	0.0346		0.0054	0.7003	0.5550	0.5263	0.1507	0.0195
**7**	0.0043	0.1549	0.1333	0.0119	0.2600	0.0054		0.0457	0.0231	0.0701	0.0004	0.1213
**8**	0.6517	0.4252	0.3203	0.6710	0.2322	0.7003	0.0457		0.9598	0.8425	0.1708	0.2901
**9**	0.4948	0.3544	0.2091	0.5667	0.1490	0.5550	0.0231	0.9598		0.8534	0.0673	0.1634
**10**	0.4831	0.5618	0.4544	0.5242	0.3321	0.5263	0.0701	0.8425	0.8534		0.1090	0.4235
**11**	0.1731	0.0114	0.0013	0.3209	0.0017	0.1507	0.0004	0.1708	0.0673	0.1090		0.0004
**12**	0.0134	0.8952	0.9803	0.0770	0.6933	0.0195	0.1213	0.2901	0.1634	0.4235	0.0004	
**13**	0.2406	0.7598	0.7911	0.2615	0.9459	0.2579	0.5063	0.4195	0.4013	0.5048	0.0798	0.7947
**14**	0.3176	0.8104	0.7243	0.3657	0.5523	0.3494	0.1429	0.6359	0.6149	0.7763	0.0675	0.7024
**15**	0.3033	0.4187	0.2411	0.4247	0.1707	0.3576	0.0253	0.8275	0.8246	0.9752	0.0252	0.1801
**16**	0.5417	0.1119	0.0688	0.6147	0.0509	0.5075	0.0095	0.4052	0.3219	0.3127	0.8157	0.0581
**17**	0.9615	0.1976	0.1059	0.9321	0.0779	0.9784	0.0125	0.7213	0.6189	0.5659	0.2601	0.0812

Least Squares Means for effect Zone_*SAF_ID(Climat) Pr > |t| for H0: LSMean(i)=LSMean(j) Dependent Variable: SOM					
**i/j**	**13**	**14**	**15**	**16**	**17**
**1**	0.2406	0.3176	0.3033	0.5417	0.9615
**2**	0.7598	0.8104	0.4187	0.1119	0.1976
**3**	0.7911	0.7243	0.2411	0.0688	0.1059
**4**	0.2615	0.3657	0.4247	0.6147	0.9321
**5**	0.9459	0.5523	0.1707	0.0509	0.0779
**6**	0.2579	0.3494	0.3576	0.5075	0.9784
**7**	0.5063	0.1429	0.0253	0.0095	0.0125
**8**	0.4195	0.6359	0.8275	0.4052	0.7213
**9**	0.4013	0.6149	0.8246	0.3219	0.6189
**10**	0.5048	0.7763	0.9752	0.3127	0.5659
**11**	0.0798	0.0675	0.0252	0.8157	0.2601

Least Squares Means for effect Zone_*SAF_ID(Climat) Pr > |t| for H0: LSMean(i)=LSMean(j) Dependent Variable: SOM					
**i/j**	**13**	**14**	**15**	**16**	**17**
**12**	0.7947	0.7024	0.1801	0.0581	0.0812
**13**		0.6559	0.4492	0.1678	0.2789
**14**	0.6559		0.7068	0.2199	0.3951
**15**	0.4492	0.7068		0.2412	0.4664
**16**	0.1678	0.2199	0.2412		0.5607
**17**	0.2789	0.3951	0.4664	0.5607	

T Grouping for LS-Means of Zone_*SAF_ID(Climat)						
LS-means with the same letter are not significantly different.						
		SOM LSMEAN	Zone_name	SAF_ID4	Climate	LSMEAN Number
	A	1.88605600	Agboville	Fallow	Z1R2	7
	A					
B	A	1.55160000	Duekoue	Fallow	Z6R2	13
B	A					
B	A	1.52025455	Abengourou	Monocrop	Z1R2	5
B	A					
B	A	1.43771152	Duekoue	AF	Z6R2	12
B	A					
B	A	1.43350600	Abengourou	AF	Z1R2	3
B	A					
B	A	1.40793333	Oume	Fallow	Z1R1	2
B	A					
B	A	1.32748000	Duekoue	Monocrop	Z6R2	14
B	A					
B	A	1.22404000	San_Pedro	Fallow	Z1R2	10
B						
B		1.21542000	Guiglo	AF	Z6R2	15
B						
B		1.16985714	San_Pedro	AF	Z1R2	9
B						
B		1.15508000	Agboville	Monocrop	Z1R2	8
B						
B		1.04850545	Agboville	AF	Z1R2	6
B						
B		1.04206222	Guiglo	Monocrop	Z6R2	17
B						
B		1.03065217	Oume	AF	Z1R1	1
B						
B		1.01716000	Abengourou	Fallow	Z1R2	4
B						
B		0.83183000	Guiglo	Fallow	Z6R2	16
B						

T Grouping for LS-Means of Zone_*SAF_ID(Climat)						
LS-means with the same letter are not significantly different.						
		SOM LSMEAN	Zone_name	SAF_ID4	Climate	LSMEAN Number
B		0.75240286	San_Pedro	Monocrop	Z1R2	11
The LINES display does not reflect all significant comparisons. The following additional pairs are significantly different: (5,6) (5,1) (5,11) (12,6) (12,1) (12,11) (3,6) (3,1) (3,11) (2,11) (15,11)						

Dependent Variable: TN
TN

Least Squares Means

Zone_name	Climate	TN LSMEAN	Standard Error	Pr > |t|	LSMEAN Number
Oume	Z1R1	Non-est	.	.	1
Abengourou	Z1R2	0.09250000	0.00611461	<.0001	2
Agboville	Z1R2	0.07132323	0.00761227	<.0001	3
San_Pedro	Z1R2	0.06976190	0.00659704	<.0001	4
Duekoue	Z6R2	0.10195960	0.01013336	<.0001	5
Guiglo	Z6R2	0.06237374	0.00756079	<.0001	6

Least Squares Means for effect Zone_name(Climate)<br>Pr > |t| for H0: LSMean(i)=LSMean(j)<br><br>Dependent Variable: TN						
i/j	1	2	3	4	5	6
1		.	.	.	.	.
2	.		0.0313	0.0123	0.4251	0.0022
3	.	0.0313		0.8770	0.0166	0.4052
4	.	0.0123	0.8770		0.0084	0.4624
5	.	0.4251	0.0166	0.0084		0.0020
6	.	0.0022	0.4052	0.4624	0.0020	

Dependent Variable: m3Al
m3Al

Least Squares Means

Zone_name	SAF_ID4	Climate	m3Al LSMEAN	Standard Error	Pr > |t|	LSMEAN Number
**Oume**	**AF**	**Z1R1**	754.10435	48.09039	<.0001	1
**Oume**	**Fallow**	**Z1R1**	794.30444	76.87780	<.0001	2
**Abengourou**	**AF**	**Z1R2**	922.06800	51.57120	<.0001	3
**Abengourou**	**Fallow**	**Z1R2**	721.64875	81.54122	<.0001	4
**Abengourou**	**Monocrop**	**Z1R2**	1056.16000	69.53859	<.0001	5
**Agboville**	**AF**	**Z1R2**	677.93682	49.17121	<.0001	6
**Agboville**	**Fallow**	**Z1R2**	723.74600	103.14240	<.0001	7
**Agboville**	**Monocrop**	**Z1R2**	761.96833	94.15570	<.0001	8
**San_Pedro**	**AF**	**Z1R2**	966.55643	61.63937	<.0001	9
**San_Pedro**	**Fallow**	**Z1R2**	946.27833	94.15570	<.0001	10
**San_Pedro**	**Monocrop**	**Z1R2**	814.78714	61.63937	<.0001	11
**Duekoue**	**AF**	**Z6R2**	1142.47636	40.14812	<.0001	12
**Duekoue**	**Fallow**	**Z6R2**	1317.82000	163.08245	<.0001	13
**Duekoue**	**Monocrop**	**Z6R2**	1046.56000	103.14240	<.0001	14
**Guiglo**	**AF**	**Z6R2**	920.08773	49.17121	<.0001	15
**Guiglo**	**Fallow**	**Z6R2**	666.24750	115.31670	<.0001	16
**Guiglo**	**Monocrop**	**Z6R2**	866.27444	76.87780	<.0001	17

Least Squares Means for effect Zone_*SAF_ID(Climat)
Pr > |t| for H0: LSMean(i)=LSMean(j)

Dependent Variable: m3Al

i/j	1	2	3	4	5	6	7	8	9	10	11	12
**1**		0.6580	0.0182	0.7321	0.0004	0.2695	0.7899	0.9408	0.0072	0.0706	0.4386	<.0001
**2**	0.6580		0.1691	0.5175	0.0123	0.2038	0.5840	0.7905	0.0820	0.2127	0.8355	<.0001
**3**	0.0182	0.1691		0.0391	0.1230	0.0007	0.0870	0.1375	0.5805	0.8218	0.1835	0.0009
**4**	0.7321	0.5175	0.0391		0.0021	0.6467	0.9873	0.7465	0.0175	0.0729	0.3633	<.0001
**5**	0.0004	0.0123	0.1230	0.0021		<.0001	0.0082	0.0128	0.3361	0.3490	0.0101	0.2837
**6**	0.2695	0.2038	0.0007	0.6467	<.0001		0.6889	0.4298	0.0003	0.0123	0.0842	<.0001

Least Squares Means for effect Zone_*SAF_ID(Climat)<br>Pr > |t| for H0: LSMean(i)=LSMean(j)<br><br>Dependent Variable: m3Al												
i/j	1	2	3	4	5	6	7	8	9	10	11	12
7	0.7899	0.5840	0.0870	0.9873	0.0082	0.6889		0.7846	0.0447	0.1127	0.4496	0.0002
8	0.9408	0.7905	0.1375	0.7465	0.0128	0.4298	0.7846		0.0706	0.1679	0.6393	0.0003
9	0.0072	0.0820	0.5805	0.0175	0.3361	0.0003	0.0447	0.0706		0.8572	0.0832	0.0177
10	0.0706	0.2127	0.8218	0.0729	0.3490	0.0123	0.1127	0.1679	0.8572		0.2441	0.0567
11	0.4386	0.8355	0.1835	0.3633	0.0101	0.0842	0.4496	0.6393	0.0832	0.2441		<.0001
12	<.0001	<.0001	0.0009	<.0001	0.2837	<.0001	0.0002	0.0003	0.0177	0.0567	<.0001	
13	0.0011	0.0041	0.0217	0.0013	0.1416	0.0002	0.0024	0.0035	0.0453	0.0499	0.0043	0.2978
14	0.0109	0.0513	0.2817	0.0143	0.9386	0.0015	0.0280	0.0429	0.5063	0.4736	0.0552	0.3872
15	0.0167	0.1697	0.9779	0.0385	0.1117	0.0006	0.0873	0.1382	0.5563	0.8055	0.1833	0.0006
16	0.4828	0.3566	0.0442	0.6953	0.0042	0.9258	0.7106	0.5210	0.0227	0.0615	0.2573	0.0001
17	0.2176	0.5088	0.5474	0.1984	0.0685	0.0404	0.2692	0.3919	0.3101	0.5112	0.6019	0.0017

Least Squares Means for effect Zone_*SAF_ID(Climat)<br>Pr > |t| for H0: LSMean(i)=LSMean(j)<br><br>Dependent Variable: m3Al					
i/j	13	14	15	16	17
1	0.0011	0.0109	0.0167	0.4828	0.2176
2	0.0041	0.0513	0.1697	0.3566	0.5088
3	0.0217	0.2817	0.9779	0.0442	0.5474
4	0.0013	0.0143	0.0385	0.6953	0.1984
5	0.1416	0.9386	0.1117	0.0042	0.0685
6	0.0002	0.0015	0.0006	0.9258	0.0404
7	0.0024	0.0280	0.0873	0.7106	0.2692
8	0.0035	0.0429	0.1382	0.5210	0.3919
9	0.0453	0.5063	0.5563	0.0227	0.3101
10	0.0499	0.4736	0.8055	0.0615	0.5112
11	0.0043	0.0552	0.1833	0.2573	0.6019
12	0.2978	0.3872	0.0006	0.0001	0.0017
13		0.1614	0.0206	0.0013	0.0131
14	0.1614		0.2697	0.0148	0.1627
15	0.0206	0.2697		0.0442	0.5561
16	0.0013	0.0148	0.0442		0.1505

Least Squares Means for effect Zone_*SAF_ID(Climat)<br>Pr > |t| for H0: LSMean(i)=LSMean(j)<br><br>Dependent Variable: m3Al					
i/j	13	14	15	16	17
17	0.0131	0.1627	0.5561	0.1505	

T Grouping for LS-Means of Zone_*SAF_ID(Climat)									
LS-means with the same letter are not significantly different.									
					m3Al LSMEAN	Zone_name	SAF_ID4	Climate	LSMEAN Number
			A		1317.82	Duekoue	Fallow	Z6R2	13
			A						
	B		A		1142.48	Duekoue	AF	Z6R2	12
	B		A						
	B		A	C	1056.16	Abengourou	Monocrop	Z1R2	5
	B		A	C					
	B	D	A	C	1046.56	Duekoue	Monocrop	Z6R2	14
	B	D		C					
	B	D	E	C	966.56	San_Pedro	AF	Z1R2	9
	B	D	E	C					
F	B	D	E	C	946.28	San_Pedro	Fallow	Z1R2	10
F		D	E	C					
F		D	E	C	922.07	Abengourou	AF	Z1R2	3
F		D	E	C					
F		D	E	C	920.09	Guiglo	AF	Z6R2	15
F		D	E	C					
F		D	E	C	866.27	Guiglo	Monocrop	Z6R2	17
F		D	E						
F		D	E		814.79	San_Pedro	Monocrop	Z1R2	11
F		D	E						
F		D	E		794.30	Oume	Fallow	Z1R1	2
F			E						
F			E		761.97	Agboville	Monocrop	Z1R2	8
F									
F					754.10	Oume	AF	Z1R1	1
F									
F					723.75	Agboville	Fallow	Z1R2	7
F									
F					721.65	Abengourou	Fallow	Z1R2	4
F									
F					677.94	Agboville	AF	Z1R2	6
F									
F					666.25	Guiglo	Fallow	Z6R2	16

T Grouping for LS-Means of Zone_*SAF_ID(Climat)					
LS-means with the same letter are not significantly different.					
	m3Al LSMEAN	Zone_name	SAF_ID4	Climate	LSMEAN Number
The LINES display does not reflect all significant comparisons. The following additional pairs are significantly different: (12,9) (10,6) (3,1) (3,4) (3,6) (3,16) (15,1) (15,4) (15,6) (15,16) (17,6)					

Dependent Variable: m3B
m3B

Least Squares Means

Zone_name	SAF_ID4	Climate	m3B LSMEAN	Standard Error	Pr > |t|	LSMEAN Number
**Oume**	**AF**	**Z1R1**	0.23000000	0.02147834	<.0001	1
**Oume**	**Fallow**	**Z1R1**	0.30444444	0.03433549	<.0001	2
**Abengourou**	**AF**	**Z1R2**	0.29600000	0.02303295	<.0001	3
**Abengourou**	**Fallow**	**Z1R2**	0.23125000	0.03641829	<.0001	4
**Abengourou**	**Monocrop**	**Z1R2**	0.30636364	0.03105762	<.0001	5
**Agboville**	**AF**	**Z1R2**	0.22227273	0.02196106	<.0001	6
**Agboville**	**Fallow**	**Z1R2**	0.38400000	0.04606590	<.0001	7
**Agboville**	**Monocrop**	**Z1R2**	0.26166667	0.04205222	<.0001	8
**San_Pedro**	**AF**	**Z1R2**	0.23857143	0.02752964	<.0001	9
**San_Pedro**	**Fallow**	**Z1R2**	0.22166667	0.04205222	<.0001	10
**San_Pedro**	**Monocrop**	**Z1R2**	0.26714286	0.02752964	<.0001	11
**Duekoue**	**AF**	**Z6R2**	0.32333333	0.01793113	<.0001	12
**Duekoue**	**Fallow**	**Z6R2**	0.35500000	0.07283658	<.0001	13
**Duekoue**	**Monocrop**	**Z6R2**	0.33600000	0.04606590	<.0001	14
**Guiglo**	**AF**	**Z6R2**	0.24136364	0.02196106	<.0001	15
**Guiglo**	**Fallow**	**Z6R2**	0.18000000	0.05150324	0.0006	16
**Guiglo**	**Monocrop**	**Z6R2**	0.22111111	0.03433549	<.0001	17

Least Squares Means for effect Zone_*SAF_ID(Climat)
Pr > |t| for H0: LSMean(i)=LSMean(j)

Dependent Variable: m3B

i/j	1	2	3	4	5	6	7	8	9	10	11	12
**1**		0.0676	0.0374	0.9764	0.0445	0.8016	0.0028	0.5033	0.8063	0.8601	0.2888	0.0010
**2**	0.0676		0.8384	0.1452	0.9670	0.0452	0.1677	0.4317	0.1361	0.1289	0.3977	0.6264
**3**	0.0374	0.8384		0.1345	0.7890	0.0216	0.0891	0.4748	0.1112	0.1227	0.4224	0.3502
**4**	0.9764	0.1452	0.1345		0.1182	0.8330	0.0100	0.5852	0.8728	0.8634	0.4327	0.0244
**5**	0.0445	0.9670	0.7890	0.1182		0.0282	0.1639	0.3936	0.1040	0.1068	0.3458	0.6366
**6**	0.8016	0.0452	0.0216	0.8330	0.0282		0.0018	0.4073	0.6440	0.9898	0.2041	0.0005

Least Squares Means for effect Zone_*SAF_ID(Climat) Pr > |t| for H0: LSMean(i)=LSMean(j) Dependent Variable: m3B												
**i/j**	**1**	**2**	**3**	**4**	**5**	**6**	**7**	**8**	**9**	**10**	**11**	**12**
**7**	0.0028	0.1677	0.0891	0.0100	0.1639	0.0018		0.0513	0.0073	0.0100	0.0306	0.2212
**8**	0.5033	0.4317	0.4748	0.5852	0.3936	0.4073	0.0513		0.6464	0.5020	0.9134	0.1789
**9**	0.8063	0.1361	0.1112	0.8728	0.1040	0.6440	0.0073	0.6464		0.7370	0.4639	0.0106
**10**	0.8601	0.1289	0.1227	0.8634	0.1068	0.9898	0.0100	0.5020	0.7370		0.3667	0.0273
**11**	0.2888	0.3977	0.4224	0.4327	0.3458	0.2041	0.0306	0.9134	0.4639	0.3667		0.0888
**12**	0.0010	0.6264	0.3502	0.0244	0.6366	0.0005	0.2212	0.1789	0.0106	0.0273	0.0888	
**13**	0.1013	0.5308	0.4408	0.1302	0.5398	0.0826	0.7369	0.2685	0.1365	0.1145	0.2606	0.6734
**14**	0.0383	0.5835	0.4383	0.0760	0.5943	0.0270	0.4621	0.2348	0.0710	0.0683	0.2010	0.7980
**15**	0.7118	0.1233	0.0876	0.8123	0.0891	0.5395	0.0057	0.6691	0.9369	0.6785	0.4650	0.0043
**16**	0.3713	0.0458	0.0411	0.4175	0.0369	0.4512	0.0035	0.2208	0.3171	0.5316	0.1373	0.0093
**17**	0.8265	0.0877	0.0716	0.8397	0.0671	0.9773	0.0051	0.4559	0.6920	0.9918	0.2969	0.0090

Least Squares Means for effect Zone_*SAF_ID(Climat) Pr > |t| for H0: LSMean(i)=LSMean(j) Dependent Variable: m3B					
**i/j**	**13**	**14**	**15**	**16**	**17**
**1**	0.1013	0.0383	0.7118	0.3713	0.8265
**2**	0.5308	0.5835	0.1233	0.0458	0.0877
**3**	0.4408	0.4383	0.0876	0.0411	0.0716
**4**	0.1302	0.0760	0.8123	0.4175	0.8397
**5**	0.5398	0.5943	0.0891	0.0369	0.0671
**6**	0.0826	0.0270	0.5395	0.4512	0.9773
**7**	0.7369	0.4621	0.0057	0.0035	0.0051
**8**	0.2685	0.2348	0.6691	0.2208	0.4559
**9**	0.1365	0.0710	0.9369	0.3171	0.6920
**10**	0.1145	0.0683	0.6785	0.5316	0.9918
**11**	0.2606	0.2010	0.4650	0.1373	0.2969
**12**	0.6734	0.7980	0.0043	0.0093	0.0090
**13**		0.8257	0.1369	0.0512	0.0980
**14**	0.8257		0.0652	0.0251	0.0469
**15**	0.1369	0.0652		0.2744	0.6198
**16**	0.0512	0.0251	0.2744		0.5074

Least Squares Means for effect Zone_*SAF_ID(Climat)<br>Pr > |t| for H0: LSMean(i)=LSMean(j)<br><br>Dependent Variable: m3B					
i/j	13	14	15	16	17
17	0.0980	0.0469	0.6198	0.5074	

T Grouping for LS-Means of Zone_*SAF_ID(Climat)						
LS-means with the same letter are not significantly different.						
		m3B LSMEAN	Zone_name	SAF_ID4	Climate	LSMEAN Number
	A	0.38400000	Agboville	Fallow	Z1R2	7
	A					
B	A	0.35500000	Duekoue	Fallow	Z6R2	13
B	A					
B	A	0.33600000	Duekoue	Monocrop	Z6R2	14
B	A					
B	A	0.32333333	Duekoue	AF	Z6R2	12
B	A					
B	A	0.30636364	Abengourou	Monocrop	Z1R2	5
B	A					
B	A	0.30444444	Oume	Fallow	Z1R1	2
B	A					
B	A	0.29600000	Abengourou	AF	Z1R2	3
B	A					
B	A	0.26714286	San_Pedro	Monocrop	Z1R2	11
B	A					
B	A	0.26166667	Agboville	Monocrop	Z1R2	8
B						
B		0.24136364	Guiglo	AF	Z6R2	15
B						
B		0.23857143	San_Pedro	AF	Z1R2	9
B						
B		0.23125000	Abengourou	Fallow	Z1R2	4
B						
B		0.23000000	Oume	AF	Z1R1	1
B						
B		0.22227273	Agboville	AF	Z1R2	6
B						
B		0.22166667	San_Pedro	Fallow	Z1R2	10
B						
B		0.22111111	Guiglo	Monocrop	Z6R2	17
B						

T Grouping for LS-Means of Zone_*SAF_ID(Climat)						
LS-means with the same letter are not significantly different.						
		m3B LSMEAN	Zone_name	SAF_ID4	Climate	LSMEAN Number
B		0.18000000	**Guiglo**	**Fallow**	**Z6R2**	16
The LINES display does not reflect all significant comparisons. The following additional pairs are significantly different: (7,11) (14,1) (14,6) (14,17) (14,16) (12,15) (12,9) (12,4) (12,1) (12,6) (12,10) (12,17) (12,16) (5,1) (5,6) (5,16) (2,6) (2,16) (3,1) (3,6) (3,16)						

Dependent Variable: m3Ca
m3Ca

Least Squares Means

Zone_name	Climate	m3Ca LSMEAN	Standard Error	Pr > |t|	LSMEAN Number
**Oume**	**Z1R1**	Non-est	.	.	1
**Abengourou**	**Z1R2**	850.48862	96.07996	<.0001	2
**Agboville**	**Z1R2**	1045.78123	119.61309	<.0001	3
**San_Pedro**	**Z1R2**	280.73133	112.82412	0.0137	4
**Duekoue**	**Z6R2**	650.06337	159.22739	<.0001	5
**Guiglo**	**Z6R2**	247.17391	118.80414	0.0388	6

Least Squares Means for effect Zone_name(Climate)<br>Pr > |t| for H0: LSMean(i)=LSMean(j)<br><br>Dependent Variable: m3Ca						
**i/j**	**1**	**2**	**3**	**4**	**5**	**6**
**1**		.	.	.	.	.
**2**	.		0.2046	0.0002	0.2825	0.0001
**3**	.	0.2046		<.0001	0.0483	<.0001
**4**	.	0.0002	<.0001		0.0599	0.8379
**5**	.	0.2825	0.0483	0.0599		0.0440
**6**	.	0.0001	<.0001	0.8379	0.0440	

Dependent Variable: m3Fe
m3Fe

Least Squares Means

Zone_name	Climate	m3Fe LSMEAN	Standard Error	Pr > |t|	LSMEAN Number
**Oume**	**Z1R1**	Non-est	.	.	1
**Abengourou**	**Z1R2**	141.858727	6.678662	<.0001	2
**Agboville**	**Z1R2**	180.268505	8.314486	<.0001	3
**San_Pedro**	**Z1R2**	176.285476	7.205596	<.0001	4
**Duekoue**	**Z6R2**	105.545980	11.068135	<.0001	5
**Guiglo**	**Z6R2**	169.276111	8.258254	<.0001	6

Least Squares Means for effect Zone_name(Climate)
Pr > |t| for H0: LSMean(i)=LSMean(j)

Dependent Variable: m3Fe

i/j	1	2	3	4	5	6
**1**		.	.	.	.	.
**2**	.		0.0004	0.0006	0.0055	0.0106
**3**	.	0.0004		0.7177	<.0001	0.3494
**4**	.	0.0006	0.7177		<.0001	0.5232
**5**	.	0.0055	<.0001	<.0001		<.0001
**6**	.	0.0106	0.3494	0.5232	<.0001	

Climate	SAF_ID4	m3Fe LSMEAN	Standard Error	Pr > |t|	LSMEAN Number
**Z1R1**	**AF**	114.258261	8.101776	<.0001	1
**Z1R1**	**Fallow**	109.202222	12.951584	<.0001	2
**Z1R2**	**AF**	175.258489	5.290894	<.0001	3
**Z1R2**	**Fallow**	179.619667	9.081514	<.0001	4
**Z1R2**	**Monocrop**	143.534553	7.428886	<.0001	5
**Z6R2**	**AF**	127.441970	5.347209	<.0001	6
**Z6R2**	**Fallow**	137.277500	16.824602	<.0001	7

Climate	SAF_ID4	m3Fe LSMEAN	Standard Error	Pr > |t|	LSMEAN Number
**Z6R2**	**Monocrop**	147.513667	10.836073	<.0001	8

Least Squares Means for effect Climate*SAF_ID4<br>Pr > |t| for H0: LSMean(i)=LSMean(j)<br><br>Dependent Variable: m3Fe								
**i/j**	**1**	**2**	**3**	**4**	**5**	**6**	**7**	**8**
**1**		0.7410	<.0001	<.0001	0.0084	0.1760	0.2192	0.0148
**2**	0.7410		<.0001	<.0001	0.0225	0.1945	0.1876	0.0244
**3**	<.0001	<.0001		0.6786	0.0006	<.0001	0.0325	0.0225
**4**	<.0001	<.0001	0.6786		0.0024	<.0001	0.0279	0.0242
**5**	0.0084	0.0225	0.0006	0.0024		0.0803	0.7341	0.7623
**6**	0.1760	0.1945	<.0001	<.0001	0.0803		0.5781	0.0983
**7**	0.2192	0.1876	0.0325	0.0279	0.7341	0.5781		0.6096
**8**	0.0148	0.0244	0.0225	0.0242	0.7623	0.0983	0.6096	

T Grouping for LS-Means of Climate*SAF_ID4<br>LS-means with the same letter are not significantly different.					
		**m3Fe LSMEAN**	**Climate**	**SAF_ID4**	**LSMEAN Number**
	A	179.620	**Z1R2**	**Fallow**	4
	A				
	A	175.258	**Z1R2**	**AF**	3
	B	147.514	**Z6R2**	**Monocrop**	8
	B				
	B	143.535	**Z1R2**	**Monocrop**	5
	B				
C	B	137.278	**Z6R2**	**Fallow**	7
C	B				
C	B	127.442	**Z6R2**	**AF**	6
C					
C		114.258	**Z1R1**	**AF**	1
C					
C		109.202	**Z1R1**	**Fallow**	2

Dependent Variable: m3K
m3K

Least Squares Means

Zone_name	Climate	m3K LSMEAN	Standard Error	Pr > |t|	LSMEAN Number
**Oume**	**Z1R1**	Non-est	.	.	1
**Abengourou**	**Z1R2**	78.3503704	8.3579707	<.0001	2
**Agboville**	**Z1R2**	54.3364269	10.0617100	<.0001	3
**San_Pedro**	**Z1R2**	40.8832634	10.0441042	<.0001	4
**Duekoue**	**Z6R2**	87.2282500	13.2874980	<.0001	5
**Guiglo**	**Z6R2**	30.0053333	11.6969897	0.0112	6

Least Squares Means for effect Zone_name(Climate)
Pr > |t| for H0: LSMean(i)=LSMean(j)

Dependent Variable: m3K

i/j	1	2	3	4	5	6
**1**		.	.	.	.	.
**2**	.		0.0681	0.0047	0.5724	0.0010
**3**	.	0.0681		0.3454	0.0501	0.1167
**4**	.	0.0047	0.3454		0.0060	0.4814
**5**	.	0.5724	0.0501	0.0060		0.0015
**6**	.	0.0010	0.1167	0.4814	0.0015	

Climate	SAF_ID4	m3K LSMEAN	Standard Error	Pr > |t|	LSMEAN Number
**Z1R1**	**AF**	53.009130	9.720030	<.0001	1
**Z1R1**	**Fallow**	124.944286	17.619051	<.0001	2
**Z1R2**	**AF**	57.607898	6.933329	<.0001	3
**Z1R2**	**Fallow**	60.914000	11.964245	<.0001	4
**Z1R2**	**Monocrop**	55.048162	8.986852	<.0001	5
**Z6R2**	**AF**	52.991542	7.293397	<.0001	6
**Z6R2**	**Fallow**	76.679167	21.277025	0.0004	7
**Z6R2**	**Monocrop**	46.179667	14.113582	0.0013	8

Least Squares Means for effect Climate*SAF_ID4<br>Pr > |t| for H0: LSMean(i)=LSMean(j)<br><br>Dependent Variable: m3K								
**i/j**	**1**	**2**	**3**	**4**	**5**	**6**	**7**	**8**
**1**		0.0005	0.7006	0.6088	0.8778	0.9988	0.3130	0.6907
**2**	0.0005		0.0005	0.0030	0.0005	0.0002	0.0824	0.0006
**3**	0.7006	0.0005		0.8113	0.8218	0.6470	0.3953	0.4684
**4**	0.6088	0.0030	0.8113		0.6955	0.5725	0.5193	0.4269
**5**	0.8778	0.0005	0.8218	0.6955		0.8592	0.3503	0.5968
**6**	0.9988	0.0002	0.6470	0.5725	0.8592		0.2938	0.6686
**7**	0.3130	0.0824	0.3953	0.5193	0.3503	0.2938		0.2339
**8**	0.6907	0.0006	0.4684	0.4269	0.5968	0.6686	0.2339	

T Grouping for LS-Means of Climate*SAF_ID4<br>LS-means with the same letter are not significantly different.					
		**m3K LSMEAN**	**Climate**	**SAF_ID4**	**LSMEAN Number**
	A	124.9443	**Z1R1**	**Fallow**	2
	A				
B	A	76.6792	**Z6R2**	**Fallow**	7
B					
B		60.9140	**Z1R2**	**Fallow**	4
B					
B		57.6079	**Z1R2**	**AF**	3
B					
B		55.0482	**Z1R2**	**Monocrop**	5
B					
B		53.0091	**Z1R1**	**AF**	1
B					
B		52.9915	**Z6R2**	**AF**	6
B					
B		46.1797	**Z6R2**	**Monocrop**	8

Note: To ensure overall protection level, only probabilities associated with pre-planned comparisons should be used.

Dependent Variable: m3Mg
m3Mg

Least Squares Means

Climate	m3Mg LSMEAN	Standard Error	Pr > |t|	LSMEAN Number
**Z1R1**	Non-est	.	.	1
**Z1R2**	131.931877	7.554309	<.0001	2
**Z6R2**	97.573071	12.080702	<.0001	3

**Least Squares Means for effect Climate Pr > |t| for H0: LSMean(i)=LSMean(j) Dependent Variable: m3Mg**			
**i/j**	**1**	**2**	**3**
**1**		.	.
**2**	.		0.0168
**3**	.	0.0168	

Dependent Variable: ExAc
ExAc

Least Squares Means

Zone_name	Climate	ExAc LSMEAN	Standard Error	Pr > |t|	LSMEAN Number
**Oume**	**Z1R1**	Non-est	.	.	1
**Abengourou**	**Z1R2**	0.41082576	0.03706864	<.0001	2
**Agboville**	**Z1R2**	0.41982828	0.04614796	<.0001	3
**San_Pedro**	**Z1R2**	0.70063492	0.03999329	<.0001	4
**Duekoue**	**Z6R2**	0.45505051	0.06143157	<.0001	5
**Guiglo**	**Z6R2**	0.49992424	0.04583586	<.0001	6

**Least Squares Means for effect Zone_name(Climate)<br>Pr > |t| for H0: LSMean(i)=LSMean(j)<br><br>Dependent Variable: ExAc**						
**i/j**	**1**	**2**	**3**	**4**	**5**	**6**
**1**		.	.	.	.	.
**2**	.		0.8793	<.0001	0.5384	0.1323
**3**	.	0.8793		<.0001	0.6472	0.2196
**4**	.	<.0001	<.0001		0.0010	0.0012
**5**	.	0.5384	0.6472	0.0010		0.5589
**6**	.	0.1323	0.2196	0.0012	0.5589	

Dependent Variable: PSI
PSI

Source	DF	Sum of Squares	Mean Square	F Value	Pr > F
**Model**	16	76277.4136	4767.3383	3.04	0.0001
**Error**	196	307795.8768	1570.3871		
**Corrected Total**	212	384073.2904			

Least Squares Means

Zone_name	Climate	PSI LSMEAN	Standard Error	Pr > |t|	LSMEAN Number
**Oume**	**Z1R1**	Non-est	.	.	1
**Abengourou**	**Z1R2**	100.923220	6.811593	<.0001	2
**Agboville**	**Z1R2**	74.666717	8.479975	<.0001	3
**San_Pedro**	**Z1R2**	113.355635	7.349015	<.0001	4
**Duekoue**	**Z6R2**	138.825778	11.288433	<.0001	5
**Guiglo**	**Z6R2**	101.888847	8.422625	<.0001	6

Least Squares Means for effect Zone_name(Climate) Pr > |t| for H0: LSMean(i)=LSMean(j) Dependent Variable: PSI						
**i/j**	**1**	**2**	**3**	**4**	**5**	**6**
**1**		.	.	.	.	.
**2**	.		0.0167	0.2162	0.0045	0.9291
**3**	.	0.0167		0.0007	<.0001	0.0238
**4**	.	0.2162	0.0007		0.0601	0.3062
**5**	.	0.0045	<.0001	0.0601		0.0094
**6**	.	0.9291	0.0238	0.3062	0.0094	

Dependent Variable: CEC
CEC

Least Squares Means

Zone_name	Climate	CEC LSMEAN	Standard Error	Pr > |t|	LSMEAN Number
**Oume**	**Z1R1**	Non-est	.	.	1
**Abengourou**	**Z1R2**	7.22859091	0.51338267	<.0001	2
**Agboville**	**Z1R2**	8.65461616	0.63912689	<.0001	3
**San_Pedro**	**Z1R2**	4.56443223	0.55878213	<.0001	4
**Duekoue**	**Z6R2**	4.59856250	0.85134884	<.0001	5
**Guiglo**	**Z6R2**	3.22640873	0.64721585	<.0001	6

Least Squares Means for effect Zone_name(Climate)<br>Pr > |t| for H0: LSMean(i)=LSMean(j)<br><br>Dependent Variable: CEC						
**i/j**	**1**	**2**	**3**	**4**	**5**	**6**
**1**		.	.	.	.	.
**2**	.		0.0835	0.0006	0.0088	<.0001
**3**	.	0.0835		<.0001	0.0002	<.0001
**4**	.	0.0006	<.0001		0.9733	0.1193
**5**	.	0.0088	0.0002	0.9733		0.2010
**6**	.	<.0001	<.0001	0.1193	0.2010	

Dependent Variable: Clay
Clay

Least Squares Means

Zone_name	Climate	Clay LSMEAN	Standard Error	Pr > |t|	LSMEAN Number
**Oume**	**Z1R1**	Non-est	.	.	1
**Abengourou**	**Z1R2**	50.1604470	2.6891683	<.0001	2
**Agboville**	**Z1R2**	43.0195657	3.3478336	<.0001	3
**San_Pedro**	**Z1R2**	52.7146825	2.9013386	<.0001	4
**Duekoue**	**Z6R2**	62.0475253	4.4565926	<.0001	5
**Guiglo**	**Z6R2**	45.0119781	3.3251920	<.0001	6

Least Squares Means for effect Zone_name(Climate)
Pr > |t| for H0: LSMean(i)=LSMean(j)

Dependent Variable: Clay

i/j	1	2	3	4	5	6
**1**		.	.	.	.	.
**2**	.		0.0979	0.5192	0.0235	0.2301
**3**	.	0.0979		0.0298	0.0008	0.6733
**4**	.	0.5192	0.0298		0.0808	0.0825
**5**	.	0.0235	0.0008	0.0808		0.0025
**6**	.	0.2301	0.6733	0.0825	0.0025	

Dependent Variable: Silt
Silt

Least Squares Means

Zone_name	Climate	Silt LSMEAN	Standard Error	Pr > |t|	LSMEAN Number
**Oume**	**Z1R1**	Non-est	.	.	1
**Abengourou**	**Z1R2**	18.0390303	0.5462372	<.0001	2
**Agboville**	**Z1R2**	16.7752929	0.6800286	<.0001	3
**San_Pedro**	**Z1R2**	15.0030159	0.5893343	<.0001	4
**Duekoue**	**Z6R2**	14.3061313	0.9052452	<.0001	5
**Guiglo**	**Z6R2**	15.6394529	0.6754295	<.0001	6

Least Squares Means for effect Zone_name(Climate)
Pr > |t| for H0: LSMean(i)=LSMean(j)

Dependent Variable: Silt

i/j	1	2	3	4	5	6
**1**		.	.	.	.	.
**2**	.		0.1490	0.0002	0.0005	0.0063
**3**	.	0.1490		0.0503	0.0304	0.2374
**4**	.	0.0002	0.0503		0.5196	0.4785
**5**	.	0.0005	0.0304	0.5196		0.2392
**6**	.	0.0063	0.2374	0.4785	0.2392	

Dependent Variable: Sand
Sand

Least Squares Means

Zone_name	Climate	Sand LSMEAN	Standard Error	Pr > |t|	LSMEAN Number
**Oume**	**Z1R1**	Non-est	.	.	1
**Abengourou**	**Z1R2**	31.8005227	2.6329830	<.0001	2
**Agboville**	**Z1R2**	40.2051414	3.2778868	<.0001	3
**San_Pedro**	**Z1R2**	32.2823016	2.8407204	<.0001	4
**Duekoue**	**Z6R2**	23.6463434	4.3634802	<.0001	5
**Guiglo**	**Z6R2**	39.3485690	3.2557182	<.0001	6

Least Squares Means for effect Zone_name(Climate) Pr > |t| for H0: LSMean(i)=LSMean(j) Dependent Variable: Sand						
**i/j**	**1**	**2**	**3**	**4**	**5**	**6**
**1**		.	.	.	.	.
**2**	.		0.0470	0.9011	0.1112	0.0730
**3**	.	0.0470		0.0693	0.0027	0.8531
**4**	.	0.9011	0.0693		0.0988	0.1036
**5**	.	0.1112	0.0027	0.0988		0.0044
**6**	.	0.0730	0.8531	0.1036	0.0044	

Dependent Variable: SSSI
SSSI

Least Squares Means

Zone_name	SAF_ID4	Climate	SSSI LSMEAN	Standard Error	Pr > |t|	LSMEAN Number
**Oume**	**AF**	**Z1R1**	1.86166958	0.14747864	<.0001	1
**Oume**	**Fallow**	**Z1R1**	2.22024363	0.23576091	<.0001	2
**Abengourou**	**AF**	**Z1R2**	2.00310067	0.15815323	<.0001	3
**Abengourou**	**Fallow**	**Z1R2**	1.51190973	0.25006221	<.0001	4
**Abengourou**	**Monocrop**	**Z1R2**	2.01144596	0.21325377	<.0001	5
**Agboville**	**AF**	**Z1R2**	1.78616949	0.15079318	<.0001	6
**Agboville**	**Fallow**	**Z1R2**	2.98402944	0.31630645	<.0001	7

Zone_name	SAF_ID4	Climate	SSSI LSMEAN	Standard Error	Pr > |t|	LSMEAN Number
**Agboville**	**Monocrop**	**Z1R2**	1.89814457	0.28874696	<.0001	8
**San_Pedro**	**AF**	**Z1R2**	1.62432755	0.18902926	<.0001	9
**San_Pedro**	**Fallow**	**Z1R2**	1.69461138	0.28874696	<.0001	10
**San_Pedro**	**Monocrop**	**Z1R2**	1.14993993	0.18902926	<.0001	11
**Duekoue**	**AF**	**Z6R2**	1.91478901	0.12312212	<.0001	12
**Duekoue**	**Fallow**	**Z6R2**	1.85806943	0.50012441	0.0003	13
**Duekoue**	**Monocrop**	**Z6R2**	1.81569984	0.31630645	<.0001	14
**Guiglo**	**AF**	**Z6R2**	1.80069366	0.15079318	<.0001	15
**Guiglo**	**Fallow**	**Z6R2**	1.53812360	0.35364136	<.0001	16
**Guiglo**	**Monocrop**	**Z6R2**	1.60255301	0.23576091	<.0001	17

Least Squares Means for effect Zone_*SAF_ID(Climat)
Pr > |t| for H0: LSMean(i)=LSMean(j)

Dependent Variable: SSSI

i/j	1	2	3	4	5	6	7	8	9	10	11	12
**1**		0.1988	0.5139	0.2297	0.5642	0.7208	0.0015	0.9105	0.3234	0.6070	0.0034	0.7825
**2**	0.1988		0.4453	0.0406	0.5121	0.1225	0.0543	0.3886	0.0500	0.1601	0.0005	0.2522
**3**	0.5139	0.4453		0.0985	0.9750	0.3221	0.0061	0.7502	0.1259	0.3499	0.0007	0.6600
**4**	0.2297	0.0406	0.0985		0.1301	0.3488	0.0003	0.3132	0.7203	0.6330	0.2496	0.1499
**5**	0.5642	0.5121	0.9750	0.1301		0.3895	0.0116	0.7526	0.1759	0.3785	0.0028	0.6951
**6**	0.7208	0.1225	0.3221	0.3488	0.3895		0.0008	0.7314	0.5041	0.7790	0.0092	0.5096
**7**	0.0015	0.0543	0.0061	0.0003	0.0116	0.0008		0.0120	0.0003	0.0029	<.0001	0.0019
**8**	0.9105	0.3886	0.7502	0.3132	0.7526	0.7314	0.0120		0.4285	0.6187	0.0314	0.9578
**9**	0.3234	0.0500	0.1259	0.7203	0.1759	0.5041	0.0003	0.4285		0.8388	0.0775	0.1994
**10**	0.6070	0.1601	0.3499	0.6330	0.3785	0.7790	0.0029	0.6187	0.8388		0.1161	0.4839
**11**	0.0034	0.0005	0.0007	0.2496	0.0028	0.0092	<.0001	0.0314	0.0775	0.1161		0.0008
**12**	0.7825	0.2522	0.6600	0.1499	0.6951	0.5096	0.0019	0.9578	0.1994	0.4839	0.0008	
**13**	0.9945	0.5132	0.7825	0.5366	0.7782	0.8907	0.0585	0.9447	0.6625	0.7774	0.1869	0.9124
**14**	0.8953	0.3064	0.5968	0.4521	0.6084	0.9329	0.0097	0.8475	0.6041	0.7777	0.0723	0.7706
**15**	0.7728	0.1354	0.3554	0.3239	0.4207	0.9458	0.0009	0.7651	0.4666	0.7450	0.0077	0.5585
**16**	0.3995	0.1101	0.2315	0.9518	0.2531	0.5196	0.0026	0.4313	0.8300	0.7321	0.3342	0.3157
**17**	0.3526	0.0654	0.1599	0.7923	0.1999	0.5125	0.0006	0.4288	0.9426	0.8052	0.1358	0.2418

Least Squares Means for effect Zone_*SAF_ID(Climat) Pr > |t| for H0: LSMean(i)=LSMean(j) Dependent Variable: SSSI					
**i/j**	**13**	**14**	**15**	**16**	**17**
**1**	0.9945	0.8953	0.7728	0.3995	0.3526
**2**	0.5132	0.3064	0.1354	0.1101	0.0654
**3**	0.7825	0.5968	0.3554	0.2315	0.1599
**4**	0.5366	0.4521	0.3239	0.9518	0.7923
**5**	0.7782	0.6084	0.4207	0.2531	0.1999
**6**	0.8907	0.9329	0.9458	0.5196	0.5125
**7**	0.0585	0.0097	0.0009	0.0026	0.0006
**8**	0.9447	0.8475	0.7651	0.4313	0.4288
**9**	0.6625	0.6041	0.4666	0.8300	0.9426
**10**	0.7774	0.7777	0.7450	0.7321	0.8052
**11**	0.1869	0.0723	0.0077	0.3342	0.1358
**12**	0.9124	0.7706	0.5585	0.3157	0.2418
**13**		0.9430	0.9126	0.6020	0.6445
**14**	0.9430		0.9659	0.5592	0.5896
**15**	0.9126	0.9659		0.4954	0.4798
**16**	0.6020	0.5592	0.4954		0.8797
**17**	0.6445	0.5896	0.4798	0.8797	

T Grouping for LS-Means of Zone_*SAF_ID(Climat)									
LS-means with the same letter are not significantly different.									
				**SSSI LSMEAN**	**Zone_name**	**SAF_ID4**	**Climate**	**LSMEAN Number**
		A		2.9840294	**Agboville**	**Fallow**	**Z1R2**	7
		A						
B		A		2.2202436	**Oume**	**Fallow**	**Z1R1**	2
B		A						
B		A	C	2.0114460	**Abengourou**	**Monocrop**	**Z1R2**	5
B		A	C					
B		A	C	2.0031007	**Abengourou**	**AF**	**Z1R2**	3
B		A	C					
B		A	C	1.9147890	**Duekoue**	**AF**	**Z6R2**	12
B		A	C					

T Grouping for LS-Means of Zone_*SAF_ID(Climat)								
LS-means with the same letter are not significantly different.								
				SSSI LSMEAN	Zone_name	SAF_ID4	Climate	LSMEAN Number
B		A	C	1.8981446	**Agboville**	**Monocrop**	**Z1R2**	8
B		A	C					
B		A	C	1.8616696	**Oume**	**AF**	**Z1R1**	1
B		A	C					
B	D	A	C	1.8580694	**Duekoue**	**Fallow**	**Z6R2**	13
B	D		C					
B	D		C	1.8156998	**Duekoue**	**Monocrop**	**Z6R2**	14
B	D		C					
B	D		C	1.8006937	**Guiglo**	**AF**	**Z6R2**	15
B	D		C					
B	D		C	1.7861695	**Agboville**	**AF**	**Z1R2**	6
B	D		C					
B	D		C	1.6946114	**San_Pedro**	**Fallow**	**Z1R2**	10
B	D		C					
B	D		C	1.6243275	**San_Pedro**	**AF**	**Z1R2**	9
B	D		C					
B	D		C	1.6025530	**Guiglo**	**Monocrop**	**Z6R2**	17
B	D		C					
B	D		C	1.5381236	**Guiglo**	**Fallow**	**Z6R2**	16
	D		C					
	D		C	1.5119097	**Abengourou**	**Fallow**	**Z1R2**	4
	D							
	D			1.1499399	**San_Pedro**	**Monocrop**	**Z1R2**	11
The LINES display does not reflect all significant comparisons. The following additional pairs are significantly different: (7,5) (7,3) (7,12) (7,8) (7,1) (15,11) (6,11)								

3.SOILDATA_0_20_cm depth

Dependent Variable: pH
pH

Least Squares Means

Zone_name	Climate	pH LSMEAN	Standard Error	Pr > |t|	LSMEAN Number
Oume	Z1R1	Non-est	.	.	1
Abengourou	Z1R2	6.00561364	0.10656022	<.0001	2
Agboville	Z1R2	6.17125253	0.13266031	<.0001	3
San_Pedro	Z1R2	5.31785714	0.11496763	<.0001	4
Duekoue	Z6R2	5.96931313	0.17659568	<.0001	5
Guiglo	Z6R2	5.27920875	0.13176312	<.0001	6

Least Squares Means for effect Zone_name(Climate)
Pr > |t| for H0: LSMean(i)=LSMean(j)

Dependent Variable: pH

i/j	1	2	3	4	5	6
1		.	.	.	.	.
2	.		0.3315	<.0001	0.8605	<.0001
3	.	0.3315		<.0001	0.3617	<.0001
4	.	<.0001	<.0001		0.0023	0.8253
5	.	0.8605	0.3617	0.0023		0.0020
6	.	<.0001	<.0001	0.8253	0.0020	

Climate	SAF_ID4	pH LSMEAN	Standard Error	Pr > |t|	LSMEAN Number
Z1R1	AF	6.22173913	0.12926646	<.0001	1
Z1R1	Fallow	7.19333333	0.20664673	<.0001	2
Z1R2	AF	5.83222078	0.08441794	<.0001	3
Z1R2	Fallow	5.71891667	0.14489851	<.0001	4
Z1R2	Monocrop	5.94358586	0.11853029	<.0001	5
Z6R2	AF	5.63606061	0.08531646	<.0001	6

Climate	SAF_ID4	pH LSMEAN	Standard Error	Pr > |t|	LSMEAN Number
**Z6R2**	**Fallow**	5.52750000	0.26844197	<.0001	7
**Z6R2**	**Monocrop**	5.70922222	0.17289306	<.0001	8

Least Squares Means for effect Climate*SAF_ID4
Pr > |t| for H0: LSMean(i)=LSMean(j)

Dependent Variable: pH

i/j	1	2	3	4	5	6	7	8
**1**		<.0001	0.0124	0.0103	0.1144	0.0002	0.0208	0.0186
**2**	<.0001		<.0001	<.0001	<.0001	<.0001	<.0001	<.0001
**3**	0.0124	<.0001		0.5001	0.4450	0.1038	0.2802	0.5234
**4**	0.0103	<.0001	0.5001		0.2315	0.6227	0.5311	0.9658
**5**	0.1144	<.0001	0.4450	0.2315		0.0365	0.1578	0.2649
**6**	0.0002	<.0001	0.1038	0.6227	0.0365		0.7003	0.7047
**7**	0.0208	<.0001	0.2802	0.5311	0.1578	0.7003		0.5699
**8**	0.0186	<.0001	0.5234	0.9658	0.2649	0.7047	0.5699	

T Grouping for LS-Means of Climate*SAF_ID4

LS-means with the same letter are not significantly different.

		pH LSMEAN	Climate	SAF_ID4	LSMEAN Number
	A	7.1933333	**Z1R1**	**Fallow**	2
	B	6.2217391	**Z1R1**	**AF**	1
	B				
C	B	5.9435859	**Z1R2**	**Monocrop**	5
C					
C		5.8322208	**Z1R2**	**AF**	3
C					
C		5.7189167	**Z1R2**	**Fallow**	4
C					
C		5.7092222	**Z6R2**	**Monocrop**	8
C					
C		5.6360606	**Z6R2**	**AF**	6
C					
C		5.5275000	**Z6R2**	**Fallow**	7

T Grouping for LS-Means of Climate*SAF_ID4				
LS-means with the same letter are not significantly different.				
	pH LSMEAN	Climate	SAF_ID4	LSMEAN Number
The LINES display does not reflect all significant comparisons. The following additional pairs are significantly different: (5,6)				

Dependent Variable: SOC
SOC

Least Squares Means

Zone_name	Climate	SOC LSMEAN	Standard Error	Pr > |t|	LSMEAN Number
**Oume**	**Z1R1**	Non-est	.	.	1
**Abengourou**	**Z1R2**	1.19669697	0.08170894	<.0001	2
**Agboville**	**Z1R2**	1.20443434	0.10172213	<.0001	3
**San_Pedro**	**Z1R2**	0.94333333	0.08815562	<.0001	4
**Duekoue**	**Z6R2**	1.38741414	0.13541118	<.0001	5
**Guiglo**	**Z6R2**	0.94671717	0.10103418	<.0001	6

Least Squares Means for effect Zone_name(Climate)
Pr > |t| for H0: LSMean(i)=LSMean(j)

Dependent Variable: SOC

i/j	1	2	3	4	5	6
**1**		.	.	.	.	.
**2**	.		0.9528	0.0363	0.2293	0.0558
**3**	.	0.9528		0.0538	0.2813	0.0738
**4**	.	0.0363	0.0538		0.0065	0.9799
**5**	.	0.2293	0.2813	0.0065		0.0098
**6**	.	0.0558	0.0738	0.9799	0.0098	

Climate	SAF_ID4	SOC LSMEAN	Standard Error	Pr > |t|	LSMEAN Number
**Z1R1**	**AF**	1.05000000	0.09911977	<.0001	1
**Z1R1**	**Fallow**	1.69777778	0.15845391	<.0001	2
**Z1R2**	**AF**	1.11190260	0.06473053	<.0001	3
**Z1R2**	**Fallow**	1.13016667	0.11110621	<.0001	4
**Z1R2**	**Monocrop**	1.10239538	0.09088742	<.0001	5
**Z6R2**	**AF**	1.18803030	0.06541951	<.0001	6
**Z6R2**	**Fallow**	1.14250000	0.20583767	<.0001	7

Climate	SAF_ID4	SOC LSMEAN	Standard Error	Pr > |t|	LSMEAN Number
Z6R2	Monocrop	1.17066667	0.13257205	<.0001	8

Least Squares Means for effect Climate*SAF_ID4<br>Pr > |t| for H0: LSMean(i)=LSMean(j)<br><br>Dependent Variable: SOC								
i/j	1	2	3	4	5	6	7	8
1		0.0006	0.6016	0.5909	0.6972	0.2466	0.6860	0.4669
2	0.0006		0.0008	0.0038	0.0013	0.0033	0.0338	0.0115
3	0.6016	0.0008		0.8872	0.9322	0.4091	0.8874	0.6908
4	0.5909	0.0038	0.8872		0.8468	0.6541	0.9580	0.8151
5	0.6972	0.0013	0.9322	0.8468		0.4454	0.8587	0.6715
6	0.2466	0.0033	0.4091	0.6541	0.4454		0.8333	0.9066
7	0.6860	0.0338	0.8874	0.9580	0.8587	0.8333		0.9085
8	0.4669	0.0115	0.6908	0.8151	0.6715	0.9066	0.9085	

T Grouping for LS-Means of Climate*SAF_ID4<br>LS-means with the same letter are not significantly different.				
	SOC LSMEAN	Climate	SAF_ID4	LSMEAN Number
A	1.6977778	Z1R1	Fallow	2
B	1.1880303	Z6R2	AF	6
B				
B	1.1706667	Z6R2	Monocrop	8
B				
B	1.1425000	Z6R2	Fallow	7
B				
B	1.1301667	Z1R2	Fallow	4
B				
B	1.1119026	Z1R2	AF	3
B				
B	1.1023954	Z1R2	Monocrop	5
B				
B	1.0500000	Z1R1	AF	1

Dependent Variable: TN
TN

Least Squares Means

Zone_name	Climate	TN LSMEAN	Standard Error	Pr > |t|	LSMEAN Number
**Oume**	**Z1R1**	Non-est	.	.	1
**Abengourou**	**Z1R2**	0.11568939	0.00763525	<.0001	2
**Agboville**	**Z1R2**	0.09449495	0.00950537	<.0001	3
**San_Pedro**	**Z1R2**	0.08793651	0.00823765	<.0001	4
**Duekoue**	**Z6R2**	0.13190909	0.01265342	<.0001	5
**Guiglo**	**Z6R2**	0.07937710	0.00944108	<.0001	6

Least Squares Means for effect Zone_name(Climate)
Pr > |t| for H0: LSMean(i)=LSMean(j)

Dependent Variable: TN

i/j	1	2	3	4	5	6
**1**		.	.	.	.	.
**2**	.		0.0837	0.0143	0.2738	0.0031
**3**	.	0.0837		0.6027	0.0191	0.2605
**4**	.	0.0143	0.6027		0.0040	0.4953
**5**	.	0.2738	0.0191	0.0040		0.0010
**6**	.	0.0031	0.2605	0.4953	0.0010	

Dependent Variable: m3Al
m3Al

Least Squares Means

Zone_name	Climate	m3Al LSMEAN	Standard Error	Pr > |t|	LSMEAN Number
**Oume**	**Z1R1**	Non-est	.	.	1
**Abengourou**	**Z1R2**	855.37897	36.77065	<.0001	2
**Agboville**	**Z1R2**	716.79725	45.77698	<.0001	3
**San_Pedro**	**Z1R2**	870.35214	39.67178	<.0001	4
**Duekoue**	**Z6R2**	1065.81518	60.93772	<.0001	5
**Guiglo**	**Z6R2**	772.46375	45.46739	<.0001	6

Least Squares Means for effect Zone_name(Climate)
Pr > |t| for H0: LSMean(i)=LSMean(j)

Dependent Variable: m3Al

i/j	1	2	3	4	5	6
**1**		.	.	.	.	.
**2**	.		0.0192	0.7822	0.0035	0.1578
**3**	.	0.0192		0.0120	<.0001	0.3893
**4**	.	0.7822	0.0120		0.0078	0.1064
**5**	.	0.0035	<.0001	0.0078		0.0002
**6**	.	0.1578	0.3893	0.1064	0.0002	

Dependent Variable: m3B
m3B

Least Squares Means

Zone_name	Climate	m3B LSMEAN	Standard Error	Pr > |t|	LSMEAN Number
Oume	Z1R1	Non-est	.	.	1
Abengourou	Z1R2	0.37150000	0.02343521	<.0001	2
Agboville	Z1R2	0.38044444	0.02917526	<.0001	3
San_Pedro	Z1R2	0.28880952	0.02528421	<.0001	4
Duekoue	Z6R2	0.42441414	0.03883773	<.0001	5
Guiglo	Z6R2	0.25817340	0.02897795	<.0001	6

Least Squares Means for effect Zone_name(Climate)
Pr > |t| for H0: LSMean(i)=LSMean(j)

Dependent Variable: m3B

i/j	1	2	3	4	5	6
1		.	.	.	.	.
2	.		0.8113	0.0174	0.2448	0.0027
3	.	0.8113		0.0186	0.3665	0.0033
4	.	0.0174	0.0186		0.0038	0.4266
5	.	0.2448	0.3665	0.0038		0.0007
6	.	0.0027	0.0033	0.4266	0.0007	

Dependent Variable: m3Ca
m3Ca

Least Squares Means

Zone_name	Climate	m3Ca LSMEAN	Standard Error	Pr > |t|	LSMEAN Number
Oume	Z1R1	Non-est	.	.	1
Abengourou	Z1R2	1135.48907	170.35167	<.0001	2
Agboville	Z1R2	1358.30492	212.07637	<.0001	3
San_Pedro	Z1R2	366.86583	190.73007	0.0559	4
Duekoue	Z6R2	1116.27174	282.31331	0.0001	5
Guiglo	Z6R2	338.59058	211.20206	0.1106	6

Least Squares Means for effect Zone_name(Climate)<br>Pr > |t| for H0: LSMean(i)=LSMean(j)<br><br>Dependent Variable: m3Ca						
i/j	1	2	3	4	5	6
1		.	.	.	.	.
2	.		0.4137	0.0030	0.9536	0.0037
3	.	0.4137		0.0006	0.4939	0.0008
4	.	0.0030	0.0006		0.0290	0.9210
5	.	0.9536	0.4939	0.0290		0.0286
6	.	0.0037	0.0008	0.9210	0.0286	

Climate	SAF_ID4	m3Ca LSMEAN	Standard Error	Pr > |t|	LSMEAN Number
Z1R1	AF	912.28000	206.65082	<.0001	1
Z1R1	Fallow	2430.40333	330.35417	<.0001	2
Z1R2	AF	916.89653	139.68470	<.0001	3
Z1R2	Fallow	937.14342	231.64087	<.0001	4
Z1R2	Monocrop	1006.61987	192.88528	<.0001	5
Z6R2	AF	747.88703	138.32520	<.0001	6
Z6R2	Fallow	620.05500	429.14266	0.1501	7
Z6R2	Monocrop	814.35144	276.39413	0.0036	8

Least Squares Means for effect Climate*SAF_ID4<br>Pr > |t| for H0: LSMean(i)=LSMean(j)<br><br>Dependent Variable: m3Ca								
i/j	1	2	3	4	5	6	7	8
1		0.0001	0.9853	0.9362	0.7389	0.5094	0.5403	0.7769
2	0.0001		<.0001	0.0003	0.0003	<.0001	0.0010	0.0002
3	0.9853	<.0001		0.9404	0.7068	0.3910	0.5115	0.7409
4	0.9362	0.0003	0.9404		0.8180	0.4839	0.5163	0.7339
5	0.7389	0.0003	0.7068	0.8180		0.2771	0.4123	0.5690
6	0.5094	<.0001	0.3910	0.4839	0.2771		0.7771	0.8300
7	0.5403	0.0010	0.5115	0.5163	0.4123	0.7771		0.7039
8	0.7769	0.0002	0.7409	0.7339	0.5690	0.8300	0.7039	

T Grouping for LS-Means of Climate*SAF_ID4				
LS-means with the same letter are not significantly different.				
	m3Ca LSMEAN	Climate	SAF_ID4	LSMEAN Number
A	2430.40	**Z1R1**	**Fallow**	2
B	1006.62	**Z1R2**	**Monocrop**	5
B				
B	937.14	**Z1R2**	**Fallow**	4
B				
B	916.90	**Z1R2**	**AF**	3
B				
B	912.28	**Z1R1**	**AF**	1
B				
B	814.35	**Z6R2**	**Monocrop**	8
B				
B	747.89	**Z6R2**	**AF**	6
B				
B	620.05	**Z6R2**	**Fallow**	7

Dependent Variable: m3Fe
m3Fe

Least Squares Means

Zone_name	Climate	m3Fe LSMEAN	Standard Error	Pr > |t|	LSMEAN Number
Oume	Z1R1	Non-est	.	.	1
Abengourou	Z1R2	155.918038	7.875994	<.0001	2
Agboville	Z1R2	174.303818	9.805083	<.0001	3
San_Pedro	Z1R2	188.380476	8.497395	<.0001	4
Duekoue	Z6R2	112.437707	13.052398	<.0001	5
Guiglo	Z6R2	195.976970	9.738770	<.0001	6

Least Squares Means for effect Zone_name(Climate) Pr > |t| for H0: LSMean(i)=LSMean(j) Dependent Variable: m3Fe						
i/j	1	2	3	4	5	6
1		.	.	.	.	.
2	.		0.1454	0.0056	0.0048	0.0016
3	.	0.1454		0.2793	0.0002	0.1184
4	.	0.0056	0.2793		<.0001	0.5574
5	.	0.0048	0.0002	<.0001		<.0001
6	.	0.0016	0.1184	0.5574	<.0001	

Climate	SAF_ID4	m3Fe LSMEAN	Standard Error	Pr > |t|	LSMEAN Number
Z1R1	AF	127.150435	9.554239	<.0001	1
Z1R1	Fallow	105.097778	15.273507	<.0001	2
Z1R2	AF	182.020294	6.239431	<.0001	3
Z1R2	Fallow	185.964917	10.709622	<.0001	4
Z1R2	Monocrop	150.617121	8.760716	<.0001	5
Z6R2	AF	146.451515	6.305842	<.0001	6
Z6R2	Fallow	143.897500	19.840868	<.0001	7
Z6R2	Monocrop	172.273000	12.778733	<.0001	8

Least Squares Means for effect Climate*SAF_ID4<br>Pr > |t| for H0: LSMean(i)=LSMean(j)<br><br>Dependent Variable: m3Fe								
**i/j**	**1**	**2**	**3**	**4**	**5**	**6**	**7**	**8**
**1**		0.2224	<.0001	<.0001	0.0718	0.0934	0.4479	0.0052
**2**	0.2224		<.0001	<.0001	0.0105	0.0131	0.1229	0.0009
**3**	<.0001	<.0001		0.7506	0.0039	<.0001	0.0683	0.4939
**4**	<.0001	<.0001	0.7506		0.0114	0.0017	0.0636	0.4125
**5**	0.0718	0.0105	0.0039	0.0114		0.7000	0.7570	0.1638
**6**	0.0934	0.0131	<.0001	0.0017	0.7000		0.9025	0.0715
**7**	0.4479	0.1229	0.0683	0.0636	0.7570	0.9025		0.2307
**8**	0.0052	0.0009	0.4939	0.4125	0.1638	0.0715	0.2307	

T Grouping for LS-Means of Climate*SAF_ID4						
**LS-means with the same letter are not significantly different.**						
			**m3Fe LSMEAN**	**Climate**	**SAF_ID4**	**LSMEAN Number**
	A		185.965	**Z1R2**	**Fallow**	4
	A					
	A		182.020	**Z1R2**	**AF**	3
	A					
	A		172.273	**Z6R2**	**Monocrop**	8
	A					
B	A		150.617	**Z1R2**	**Monocrop**	5
B	A					
B	A		146.452	**Z6R2**	**AF**	6
B	A					
B	A	C	143.897	**Z6R2**	**Fallow**	7
B		C				
B		C	127.150	**Z1R1**	**AF**	1
		C				
		C	105.098	**Z1R1**	**Fallow**	2
**The LINES display does not reflect all significant comparisons. The following additional pairs are significantly different: (4,5) (4,6) (3,5) (3,6)**						

Dependent Variable: m3K
m3K

Least Squares Means

Zone_name	Climate	m3K LSMEAN	Standard Error	Pr > |t|	LSMEAN Number
**Oume**	**Z1R1**	Non-est	.	.	1
**Abengourou**	**Z1R2**	100.370386	12.480823	<.0001	2
**Agboville**	**Z1R2**	67.503606	15.537785	<.0001	3
**San_Pedro**	**Z1R2**	53.745608	14.285175	0.0002	4
**Duekoue**	**Z6R2**	121.906576	20.683697	<.0001	5
**Guiglo**	**Z6R2**	43.139926	15.518732	0.0060	6

Least Squares Means for effect Zone_name(Climate)
Pr > |t| for H0: LSMean(i)=LSMean(j)

Dependent Variable: m3K

i/j	1	2	3	4	5	6
**1**		.	.	.	.	.
**2**	.		0.1008	0.0149	0.3738	0.0045
**3**	.	0.1008		0.5153	0.0368	0.2686
**4**	.	0.0149	0.5153		0.0073	0.6157
**5**	.	0.3738	0.0368	0.0073		0.0026
**6**	.	0.0045	0.2686	0.6157	0.0026	

Climate	SAF_ID4	m3K LSMEAN	Standard Error	Pr > |t|	LSMEAN Number
**Z1R1**	**AF**	73.660476	15.844853	<.0001	1
**Z1R1**	**Fallow**	196.385556	24.203413	<.0001	2
**Z1R2**	**AF**	75.954952	10.048866	<.0001	3
**Z1R2**	**Fallow**	73.682917	17.537039	<.0001	4
**Z1R2**	**Monocrop**	71.981732	13.882811	<.0001	5
**Z6R2**	**AF**	79.978614	10.288068	<.0001	6
**Z6R2**	**Fallow**	85.331250	31.441156	0.0073	7

Climate	SAF_ID4	m3K LSMEAN	Standard Error	Pr > |t|	LSMEAN Number
**Z6R2**	**Monocrop**	82.259889	20.250028	<.0001	8

**Least Squares Means for effect Climate*SAF_ID4<br>Pr > |t| for H0: LSMean(i)=LSMean(j)<br><br>Dependent Variable: m3K**								
**i/j**	**1**	**2**	**3**	**4**	**5**	**6**	**7**	**8**
**1**		<.0001	0.9028	0.9992	0.9366	0.7384	0.7406	0.7384
**2**	<.0001		<.0001	<.0001	<.0001	<.0001	0.0057	0.0004
**3**	0.9028	<.0001		0.9106	0.8169	0.7799	0.7767	0.7806
**4**	0.9992	<.0001	0.9106		0.9395	0.7572	0.7466	0.7492
**5**	0.9366	<.0001	0.8169	0.9395		0.6440	0.6981	0.6760
**6**	0.7384	<.0001	0.7799	0.7572	0.6440		0.8716	0.9201
**7**	0.7406	0.0057	0.7767	0.7466	0.6981	0.8716		0.9346
**8**	0.7384	0.0004	0.7806	0.7492	0.6760	0.9201	0.9346	

**T Grouping for LS-Means of Climate*SAF_ID4**				
**LS-means with the same letter are not significantly different.**				
	**m3K LSMEAN**	**Climate**	**SAF_ID4**	**LSMEAN Number**
A	196.3856	**Z1R1**	**Fallow**	2
B	85.3312	**Z6R2**	**Fallow**	7
B				
B	82.2599	**Z6R2**	**Monocrop**	8
B				
B	79.9786	**Z6R2**	**AF**	6
B				
B	75.9550	**Z1R2**	**AF**	3
B				
B	73.6829	**Z1R2**	**Fallow**	4
B				
B	73.6605	**Z1R1**	**AF**	1
B				
B	71.9817	**Z1R2**	**Monocrop**	5

Dependent Variable: m3Mg
m3Mg

Least Squares Means

Zone_name	Climate	m3Mg LSMEAN	Standard Error	Pr > |t|	LSMEAN Number
**Oume**	**Z1R1**	Non-est	.	.	1
**Abengourou**	**Z1R2**	184.163311	14.904361	<.0001	2
**Agboville**	**Z1R2**	178.380111	18.603590	<.0001	3
**San_Pedro**	**Z1R2**	118.946032	16.080288	<.0001	4
**Duekoue**	**Z6R2**	157.147010	24.700078	<.0001	5
**Guiglo**	**Z6R2**	96.878342	18.429439	<.0001	6

Least Squares Means for effect Zone_name(Climate)
Pr > |t| for H0: LSMean(i)=LSMean(j)

Dependent Variable: m3Mg

i/j	1	2	3	4	5	6
**1**		.	.	.	.	.
**2**	.		0.8086	0.0033	0.3502	0.0003
**3**	.	0.8086		0.0166	0.4931	0.0021
**4**	.	0.0033	0.0166		0.1965	0.3680
**5**	.	0.3502	0.4931	0.1965		0.0519
**6**	.	0.0003	0.0021	0.3680	0.0519	

Climate	SAF_ID4	m3Mg LSMEAN	Standard Error	Pr > |t|	LSMEAN Number
**Z1R1**	**AF**	152.965217	18.080237	<.0001	1
**Z1R1**	**Fallow**	266.688889	28.903257	<.0001	2
**Z1R2**	**AF**	157.886516	11.883690	<.0001	3
**Z1R2**	**Fallow**	160.809972	20.266659	<.0001	4
**Z1R2**	**Monocrop**	162.792965	16.578590	<.0001	5
**Z6R2**	**AF**	132.095833	11.933039	<.0001	6
**Z6R2**	**Fallow**	116.713750	37.546432	0.0022	7

Climate	SAF_ID4	m3Mg LSMEAN	Standard Error	Pr > |t|	LSMEAN Number
**Z6R2**	**Monocrop**	132.228444	24.182200	<.0001	8

Least Squares Means for effect Climate*SAF_ID4<br>Pr > |t| for H0: LSMean(i)=LSMean(j)<br><br>Dependent Variable: m3Mg								
**i/j**	**1**	**2**	**3**	**4**	**5**	**6**	**7**	**8**
**1**		0.0010	0.8203	0.7730	0.6891	0.3366	0.3854	0.4930
**2**	0.0010		0.0006	0.0031	0.0021	<.0001	0.0018	0.0005
**3**	0.8203	0.0006		0.9011	0.8102	0.1273	0.2971	0.3421
**4**	0.7730	0.0031	0.9011		0.9397	0.2236	0.3027	0.3661
**5**	0.6891	0.0021	0.8102	0.9397		0.1345	0.2630	0.2985
**6**	0.3366	<.0001	0.1273	0.2236	0.1345		0.6966	0.9961
**7**	0.3854	0.0018	0.2971	0.3027	0.2630	0.6966		0.7287
**8**	0.4930	0.0005	0.3421	0.3661	0.2985	0.9961	0.7287	

T Grouping for LS-Means of Climate*SAF_ID4<br>LS-means with the same letter are not significantly different.				
	**m3Mg LSMEAN**	**Climate**	**SAF_ID4**	**LSMEAN Number**
A	266.689	**Z1R1**	**Fallow**	2
B	162.793	**Z1R2**	**Monocrop**	5
B				
B	160.810	**Z1R2**	**Fallow**	4
B				
B	157.887	**Z1R2**	**AF**	3
B				
B	152.965	**Z1R1**	**AF**	1
B				
B	132.228	**Z6R2**	**Monocrop**	8
B				
B	132.096	**Z6R2**	**AF**	6
B				
B	116.714	**Z6R2**	**Fallow**	7

Dependent Variable: ExAc
ExAc

Least Squares Means

Zone_name	Climate	ExAc LSMEAN	Standard Error	Pr > |t|	LSMEAN Number
**Oume**	**Z1R1**	Non-est	.	.	1
**Abengourou**	**Z1R2**	0.37542424	0.02871098	<.0001	2
**Agboville**	**Z1R2**	0.40191919	0.03574324	<.0001	3
**San_Pedro**	**Z1R2**	0.59000000	0.03097622	<.0001	4
**Duekoue**	**Z6R2**	0.35222222	0.04758094	<.0001	5
**Guiglo**	**Z6R2**	0.54272727	0.03550151	<.0001	6

Least Squares Means for effect Zone_name(Climate)
Pr > |t| for H0: LSMean(i)=LSMean(j)

Dependent Variable: ExAc

i/j	1	2	3	4	5	6
**1**		.	.	.	.	.
**2**	.		0.5640	<.0001	0.6768	0.0003
**3**	.	0.5640		<.0001	0.4047	0.0057
**4**	.	<.0001	<.0001		<.0001	0.3169
**5**	.	0.6768	0.4047	<.0001		0.0016
**6**	.	0.0003	0.0057	0.3169	0.0016	

Dependent Variable: PSI
PSI

Least Squares Means

Zone_name	Climate	PSI LSMEAN	Standard Error	Pr > |t|	LSMEAN Number
**Oume**	**Z1R1**	Non-est	.	.	1
**Abengourou**	**Z1R2**	81.5381818	6.3368616	<.0001	2
**Agboville**	**Z1R2**	59.0144040	7.8889665	<.0001	3
**San_Pedro**	**Z1R2**	94.1018254	6.8368281	<.0001	4
**Duekoue**	**Z6R2**	94.2313939	10.5016897	<.0001	5
**Guiglo**	**Z6R2**	73.5847391	7.8356129	<.0001	6

Least Squares Means for effect Zone_name(Climate)
Pr > |t| for H0: LSMean(i)=LSMean(j)

Dependent Variable: PSI

i/j	1	2	3	4	5	6
**1**		.	.	.	.	.
**2**	.		0.0272	0.1793	0.3020	0.4309
**3**	.	0.0272		0.0009	0.0080	0.1916
**4**	.	0.1793	0.0009		0.9918	0.0499
**5**	.	0.3020	0.0080	0.9918		0.1167
**6**	.	0.4309	0.1916	0.0499	0.1167	

Dependent Variable: CEC
CEC

Least Squares Means

Zone_name	Climate	CEC LSMEAN	Standard Error	Pr > |t|	LSMEAN Number
**Oume**	**Z1R1**	Non-est	.	.	1
**Abengourou**	**Z1R2**	9.1860303	0.7557004	<.0001	2
**Agboville**	**Z1R2**	10.1399091	0.9407961	<.0001	3

Zone_name	Climate	CEC LSMEAN	Standard Error	Pr > |t|	LSMEAN Number
San_Pedro	Z1R2	5.7615079	0.8153237	<.0001	4
Duekoue	Z6R2	7.7990505	1.2523756	<.0001	5
Guiglo	Z6R2	4.0070455	0.9344335	<.0001	6

Least Squares Means for effect Zone_name(Climate) Pr > |t| for H0: LSMean(i)=LSMean(j) Dependent Variable: CEC						
i/j	1	2	3	4	5	6
1		.	.	.	.	.
2	.		0.4302	0.0024	0.3442	<.0001
3	.	0.4302		0.0005	0.1367	<.0001
4	.	0.0024	0.0005		0.1743	0.1587
5	.	0.3442	0.1367	0.1743		0.0161
6	.	<.0001	<.0001	0.1587	0.0161	

Climate	SAF_ID4	CEC LSMEAN	Standard Error	Pr > |t|	LSMEAN Number
Z1R1	AF	7.5026087	0.9167277	<.0001	1
Z1R1	Fallow	14.2611111	1.4654906	<.0001	2
Z1R2	AF	8.3284329	0.5986724	<.0001	3
Z1R2	Fallow	8.2728889	1.0275866	<.0001	4
Z1R2	Monocrop	8.4861255	0.8405893	<.0001	5
Z6R2	AF	5.8218939	0.6050445	<.0001	6
Z6R2	Fallow	5.4812500	1.9037281	0.0044	7
Z6R2	Monocrop	6.4060000	1.2261174	<.0001	8

Least Squares Means for effect Climate*SAF_ID4 Pr > |t| for H0: LSMean(i)=LSMean(j) Dependent Variable: CEC								
i/j	1	2	3	4	5	6	7	8
1		0.0001	0.4516	0.5766	0.4300	0.1276	0.3399	0.4747
2	0.0001		0.0002	0.0010	0.0008	<.0001	0.0003	<.0001
3	0.4516	0.0002		0.9628	0.8787	0.0036	0.1553	0.1604
4	0.5766	0.0010	0.9628		0.8726	0.0412	0.1984	0.2446
5	0.4300	0.0008	0.8787	0.8726		0.0108	0.1504	0.1633

Least Squares Means for effect Climate*SAF_ID4<br>Pr > |t| for H0: LSMean(i)=LSMean(j)<br><br>Dependent Variable: CEC								
**i/j**	**1**	**2**	**3**	**4**	**5**	**6**	**7**	**8**
**6**	0.1276	<.0001	0.0036	0.0412	0.0108		0.8648	0.6697
**7**	0.3399	0.0003	0.1553	0.1984	0.1504	0.8648		0.6834
**8**	0.4747	<.0001	0.1604	0.2446	0.1633	0.6697	0.6834	

T Grouping for LS-Means of Climate*SAF_ID4				
**LS-means with the same letter are not significantly different.**				
	**CEC LSMEAN**	**Climate**	**SAF_ID4**	**LSMEAN Number**
A	14.261111	**Z1R1**	**Fallow**	2
B	8.486126	**Z1R2**	**Monocrop**	5
B				
B	8.328433	**Z1R2**	**AF**	3
B				
B	8.272889	**Z1R2**	**Fallow**	4
B				
B	7.502609	**Z1R1**	**AF**	1
B				
B	6.406000	**Z6R2**	**Monocrop**	8
B				
B	5.821894	**Z6R2**	**AF**	6
B				
B	5.481250	**Z6R2**	**Fallow**	7
**The LINES display does not reflect all significant comparisons. The following additional pairs are significantly different: (5,6) (3,6) (4,6)**				

Dependent Variable: Clay
Clay

Least Squares Means

Zone_name	Climate	Clay LSMEAN	Standard Error	Pr > |t|	LSMEAN Number
**Oume**	**Z1R1**	Non-est	.	.	1
**Abengourou**	**Z1R2**	45.8239318	2.4225269	<.0001	2
**Agboville**	**Z1R2**	41.1645051	3.0158831	<.0001	3
**San_Pedro**	**Z1R2**	49.6435714	2.6136597	<.0001	4
**Duekoue**	**Z6R2**	52.9950101	4.0147044	<.0001	5
**Guiglo**	**Z6R2**	40.3080303	2.9954865	<.0001	6

Least Squares Means for effect Zone_name(Climate)
Pr > |t| for H0: LSMean(i)=LSMean(j)

Dependent Variable: Clay

i/j	1	2	3	4	5	6
**1**		.	.	.	.	.
**2**	.		0.2298	0.2851	0.1278	0.1538
**3**	.	0.2298		0.0349	0.0195	0.8405
**4**	.	0.2851	0.0349		0.4850	0.0199
**5**	.	0.1278	0.0195	0.4850		0.0121
**6**	.	0.1538	0.8405	0.0199	0.0121	

Dependent Variable: Silt
Silt

Least Squares Means

Zone_name	SAF_ID4	Climate	Silt LSMEAN	Standard Error	Pr > |t|	LSMEAN Number
**Oume**	**AF**	**Z1R1**	16.0434783	0.6358930	<.0001	1
**Oume**	**Fallow**	**Z1R1**	16.8177778	1.0165453	<.0001	2
**Abengourou**	**AF**	**Z1R2**	20.8405000	0.6819193	<.0001	3
**Abengourou**	**Fallow**	**Z1R2**	19.7475000	1.0782091	<.0001	4
**Abengourou**	**Monocrop**	**Z1R2**	21.4054545	0.9194998	<.0001	5
**Agboville**	**AF**	**Z1R2**	19.8400000	0.6501845	<.0001	6
**Agboville**	**Fallow**	**Z1R2**	17.4520000	1.3638386	<.0001	7
**Agboville**	**Monocrop**	**Z1R2**	14.4016667	1.2450086	<.0001	8
**San_Pedro**	**AF**	**Z1R2**	15.9850000	0.8150494	<.0001	9

Zone_name	SAF_ID4	Climate	Silt LSMEAN	Standard Error	Pr > |t|	LSMEAN Number
San_Pedro	Fallow	Z1R2	17.5033333	1.2450086	<.0001	10
San_Pedro	Monocrop	Z1R2	16.5857143	0.8150494	<.0001	11
Duekoue	AF	Z6R2	18.2596970	0.5308734	<.0001	12
Duekoue	Fallow	Z6R2	16.9250000	2.1564181	<.0001	13
Duekoue	Monocrop	Z6R2	18.3160000	1.3638386	<.0001	14
Guiglo	AF	Z6R2	17.7495455	0.6501845	<.0001	15
Guiglo	Fallow	Z6R2	17.1400000	1.5248179	<.0001	16
Guiglo	Monocrop	Z6R2	17.5833333	1.0165453	<.0001	17

Least Squares Means for effect Zone_*SAF_ID(Climat)
Pr > |t| for H0: LSMean(i)=LSMean(j)

Dependent Variable: Silt

i/j	1	2	3	4	5	6	7	8	9	10	11	12
1		0.5192	<.0001	0.0035	<.0001	<.0001	0.3504	0.2417	0.9549	0.2977	0.6005	0.0081
2	0.5192		0.0012	0.0494	0.0010	0.0131	0.7097	0.1344	0.5235	0.6702	0.8588	0.2101
3	<.0001	0.0012		0.3926	0.6222	0.2896	0.0274	<.0001	<.0001	0.0197	<.0001	0.0032
4	0.0035	0.0494	0.3926		0.2434	0.9415	0.1883	0.0014	0.0059	0.1746	0.0203	0.2172
5	<.0001	0.0010	0.6222	0.2434		0.1661	0.0172	<.0001	<.0001	0.0125	0.0001	0.0034
6	<.0001	0.0131	0.2896	0.9415	0.1661		0.1156	0.0001	0.0003	0.0978	0.0021	0.0612
7	0.3504	0.7097	0.0274	0.1883	0.0172	0.1156		0.1002	0.3570	0.9779	0.5862	0.5817
8	0.2417	0.1344	<.0001	0.0014	<.0001	0.0001	0.1002		0.2886	0.0797	0.1438	0.0048
9	0.9549	0.5235	<.0001	0.0059	<.0001	0.0003	0.3570	0.2886		0.3088	0.6028	0.0204
10	0.2977	0.6702	0.0197	0.1746	0.0125	0.0978	0.9779	0.0797	0.3088		0.5382	0.5769
11	0.6005	0.8588	<.0001	0.0203	0.0001	0.0021	0.5862	0.1438	0.6028	0.5382		0.0868
12	0.0081	0.2101	0.0032	0.2172	0.0034	0.0612	0.5817	0.0048	0.0204	0.5769	0.0868	
13	0.6954	0.9642	0.0850	0.2431	0.0574	0.1971	0.8366	0.3121	0.6839	0.8166	0.8831	0.5485
14	0.1326	0.3795	0.0994	0.4113	0.0618	0.3144	0.6547	0.0353	0.1439	0.6604	0.2775	0.9694
15	0.0622	0.4409	0.0012	0.1142	0.0014	0.0241	0.8441	0.0181	0.0922	0.8610	0.2657	0.5440
16	0.5077	0.8606	0.0279	0.1642	0.0175	0.1050	0.8789	0.1658	0.5049	0.8538	0.7489	0.4888
17	0.2006	0.5950	0.0084	0.1458	0.0058	0.0630	0.9385	0.0492	0.2214	0.9604	0.4448	0.5560

Least Squares Means for effect Zone_*SAF_ID(Climat) Pr > |t| for H0: LSMean(i)=LSMean(j) Dependent Variable: Silt					
**i/j**	**13**	**14**	**15**	**16**	**17**
**1**	0.6954	0.1326	0.0622	0.5077	0.2006
**2**	0.9642	0.3795	0.4409	0.8606	0.5950
**3**	0.0850	0.0994	0.0012	0.0279	0.0084
**4**	0.2431	0.4113	0.1142	0.1642	0.1458
**5**	0.0574	0.0618	0.0014	0.0175	0.0058
**6**	0.1971	0.3144	0.0241	0.1050	0.0630
**7**	0.8366	0.6547	0.8441	0.8789	0.9385
**8**	0.3121	0.0353	0.0181	0.1658	0.0492
**9**	0.6839	0.1439	0.0922	0.5049	0.2214
**10**	0.8166	0.6604	0.8610	0.8538	0.9604
**11**	0.8831	0.2775	0.2657	0.7489	0.4448
**12**	0.5485	0.9694	0.5440	0.4888	0.5560
**13**		0.5863	0.7147	0.9352	0.7827
**14**	0.5863		0.7081	0.5661	0.6671
**15**	0.7147	0.7081		0.7135	0.8906
**16**	0.9352	0.5661	0.7135		0.8091
**17**	0.7827	0.6671	0.8906	0.8091	

T Grouping for LS-Means of Zone_*SAF_ID(Climat)							
LS-means with the same letter are not significantly different.							
			**Silt LSMEAN**	**Zone_name**	**SAF_ID4**	**Climate**	**LSMEAN Number**
	A		21.40545	**Abengourou**	**Monocrop**	**Z1R2**	5
	A						
	A		20.84050	**Abengourou**	**AF**	**Z1R2**	3
	A						
	A		19.84000	**Agboville**	**AF**	**Z1R2**	6
	A						
	A		19.74750	**Abengourou**	**Fallow**	**Z1R2**	4
	A						
B	A		18.31600	**Duekoue**	**Monocrop**	**Z6R2**	14
B	A						

T Grouping for LS-Means of Zone_*SAF_ID(Climat)							
LS-means with the same letter are not significantly different.							
			Silt LSMEAN	Zone_name	SAF_ID4	Climate	LSMEAN Number
B	A		18.25970	**Duekoue**	**AF**	**Z6R2**	12
B	A						
B	A		17.74955	**Guiglo**	**AF**	**Z6R2**	15
B	A						
B	A		17.58333	**Guiglo**	**Monocrop**	**Z6R2**	17
B	A						
B	A	C	17.50333	**San_Pedro**	**Fallow**	**Z1R2**	10
B	A	C					
B	A	C	17.45200	**Agboville**	**Fallow**	**Z1R2**	7
B	A	C					
B	A	C	17.14000	**Guiglo**	**Fallow**	**Z6R2**	16
B	A	C					
B	A	C	16.92500	**Duekoue**	**Fallow**	**Z6R2**	13
B		C					
B		C	16.81778	**Oume**	**Fallow**	**Z1R1**	2
B		C					
B		C	16.58571	**San_Pedro**	**Monocrop**	**Z1R2**	11
B		C					
B		C	16.04348	**Oume**	**AF**	**Z1R1**	1
B		C					
B		C	15.98500	**San_Pedro**	**AF**	**Z1R2**	9
		C					
		C	14.40167	**Agboville**	**Monocrop**	**Z1R2**	8
The LINES display does not reflect all significant comparisons. The following additional pairs are significantly different: (5,12) (5,15) (5,17) (5,10) (5,7) (5,16) (3,12) (3,15) (3,17) (3,10) (3,7) (3,16) (6,15) (12,1) (12,9)							

Dependent Variable: Sand
Sand

Least Squares Means

Zone_name	Climate	Sand LSMEAN	Standard Error	Pr > |t|	LSMEAN Number
**Oume**	**Z1R1**	Non-est	.	.	1
**Abengourou**	**Z1R2**	33.5115833	2.2809400	<.0001	2
**Agboville**	**Z1R2**	41.6042727	2.8396169	<.0001	3
**San_Pedro**	**Z1R2**	33.6650794	2.4609019	<.0001	4
**Duekoue**	**Z6R2**	29.1714242	3.7800611	<.0001	5
**Guiglo**	**Z6R2**	42.2010101	2.8204124	<.0001	6

Least Squares Means for effect Zone_name(Climate)
Pr > |t| for H0: LSMean(i)=LSMean(j)

Dependent Variable: Sand

i/j	1	2	3	4	5	6
**1**		.	.	.	.	.
**2**	.		0.0274	0.9636	0.3268	0.0175
**3**	.	0.0274		0.0359	0.0092	0.8816
**4**	.	0.9636	0.0359		0.3204	0.0237
**5**	.	0.3268	0.0092	0.3204		0.0063
**6**	.	0.0175	0.8816	0.0237	0.0063	

Dependent Variable: SSSI
SSSI

Least Squares Means

Zone_name	Climate	SSSI LSMEAN	Standard Error	Pr > |t|	LSMEAN Number
**Oume**	**Z1R1**	Non-est	.	.	1
**Abengourou**	**Z1R2**	3.00554364	0.15997097	<.0001	2

Zone_name	Climate	SSSI LSMEAN	Standard Error	Pr > |t|	LSMEAN Number
Agboville	Z1R2	3.49202036	0.19915310	<.0001	3
San_Pedro	Z1R2	2.44849378	0.17259238	<.0001	4
Duekoue	Z6R2	3.29524111	0.26511002	<.0001	5
Guiglo	Z6R2	2.81285842	0.19780622	<.0001	6

Least Squares Means for effect Zone_name(Climate)
Pr > |t| for H0: LSMean(i)=LSMean(j)

Dependent Variable: SSSI

i/j	1	2	3	4	5	6
1		.	.	.	.	.
2	.		0.0583	0.0189	0.3506	0.4497
3	.	0.0583		0.0001	0.5536	0.0165
4	.	0.0189	0.0001		0.0081	0.1667
5	.	0.3506	0.5536	0.0081		0.1463
6	.	0.4497	0.0165	0.1667	0.1463	

Climate	SAF_ID4	SSSI LSMEAN	Standard Error	Pr > |t|	LSMEAN Number
Z1R1	AF	3.35810631	0.19405817	<.0001	1
Z1R1	Fallow	4.63395984	0.31022343	<.0001	2
Z1R2	AF	2.98061717	0.12673040	<.0001	3
Z1R2	Fallow	3.00637008	0.21752540	<.0001	4
Z1R2	Monocrop	2.95907054	0.17794074	<.0001	5
Z6R2	AF	3.06413309	0.12807928	<.0001	6
Z6R2	Fallow	3.02105671	0.40299206	<.0001	7
Z6R2	Monocrop	3.07695948	0.25955154	<.0001	8

Least Squares Means for effect Climate*SAF_ID4
Pr > |t| for H0: LSMean(i)=LSMean(j)

Dependent Variable: SSSI

i/j	1	2	3	4	5	6	7	8
1		0.0006	0.1050	0.2290	0.1312	0.2076	0.4520	0.3867
2	0.0006		<.0001	<.0001	<.0001	<.0001	0.0018	0.0002
3	0.1050	<.0001		0.9186	0.9215	0.6435	0.9238	0.7391

Least Squares Means for effect Climate*SAF_ID4<br>Pr > |t| for H0: LSMean(i)=LSMean(j)<br><br>Dependent Variable: SSSI								
**i/j**	**1**	**2**	**3**	**4**	**5**	**6**	**7**	**8**
**4**	0.2290	<.0001	0.9186		0.8665	0.8192	0.9744	0.8351
**5**	0.1312	<.0001	0.9215	0.8665		0.6323	0.8882	0.7083
**6**	0.2076	<.0001	0.6435	0.8192	0.6323		0.9190	0.9647
**7**	0.4520	0.0018	0.9238	0.9744	0.8882	0.9190		0.9073
**8**	0.3867	0.0002	0.7391	0.8351	0.7083	0.9647	0.9073	

T Grouping for LS-Means of Climate*SAF_ID4				
**LS-means with the same letter are not significantly different.**				
	**SSSI LSMEAN**	**Climate**	**SAF_ID4**	**LSMEAN Number**
A	4.6339598	**Z1R1**	**Fallow**	2
B	3.3581063	**Z1R1**	**AF**	1
B				
B	3.0769595	**Z6R2**	**Monocrop**	8
B				
B	3.0641331	**Z6R2**	**AF**	6
B				
B	3.0210567	**Z6R2**	**Fallow**	7
B				
B	3.0063701	**Z1R2**	**Fallow**	4
B				
B	2.9806172	**Z1R2**	**AF**	3
B				
B	2.9590705	**Z1R2**	**Monocrop**	5
